# Supplementary material for: Graphlets in Multiplex Networks
Source: Sci Rep. 2020 Feb 5;10:1928. doi: 10.1038/s41598-020-57609-3 (PMC7002705; doi:10.1038/s41598-020-57609-3)
Supplement: Supplementary file 1 — Supplementary information [file 41598_2020_57609_MOESM1_ESM.pdf]

Supplementary Information towards the paper "Graphlets in Multiplex Networks"

Tamara Dimitrova[1,3,+], **Kristijan Petrovski**[1,3,+], **and Ljupcho Kocarev**[1,2,3,*]

[1]Macedonian Academy of Sciences and Arts, Skopje, Republic of Macedonia
[2]Faculty of Computer Science and Engineering, UKIM, Skopje, Republic of Macedonia
[3]IKT-Labs, Skopje, Macedonia
[*]lkocarev@manu.edu.mk
[+]these authors contributed equally to this work

ABSTRACT

We develop graphlet analysis for multiplex networks and discuss how this analysis can be extended to multilayer and multilevel networks as well as to graphs with node and/or link categorical attributes. The analysis has been adapted for two typical examples of multiplexes: economic trade data represented as a 957-plex network and 75 social networks each represented as a 12-plex network. We show that wedges (open triads) occur more often in economic trade networks than in social networks, indicating the tendency of a country to produce/trade of a product in local structure of triads which are not closed. Moreover, our analysis provides evidence that that the counties with small diversity tend to form correlated triangles. Wedges also appear in the social networks, however the dominant graphlets in social networks are triangles (closed triads). If the multiplex is the indicator of the strong tie, the graphlet analysis provides another evidence for the concepts of strong/weak ties and structural holes. In contrast to Granovetter's seminal work on the strength of weak ties, in which it has been documented that the wedges with only strong ties are absent, here we show that the wedges with only strong ties are not only present but also significantly correlated.

Method Description

A multilayer or multiplex network in plain terms is a normal network with the addition of link categorical attributes. These attributes create a set of possible links allowed between nodes. To start, we can consider a multilayer network with d layers. In this case a link can be in one of the possible d layers. In addition a link can connect any two layers. In summation a link can be of $d+\binom{d}{2}$ types.

Compared to this, a multiplex network contains d plexes. A link can be in one of the d plexes, or it can be in any number of plexes together. Meaning that in this case a link can be in one of 2^d-1 types. In other words links belong to the set E_t, which is defined as the power set of $\mathcal{R}=\{1,\dots,D\}$, without the empty set, $E_t=\mathcal{P}(\mathcal{R})\setminus\emptyset$. Defined in this way multiplex networks will have an exponential number of different sub-orbits. For this reason we can apply some reduction(transformation) of the set E_t. In the main text we further explore two possible reductions (plex-count,plex-count with distinct links inside orbits). In general the method remains the same with a change of the E_t (link attribute set) used.

A similar consideration can be done for directed networks, however in this paper we focus only on the multiplex case. With the addition of these link states we expand the possible states a graphlet orbit can take. Each of these states we define as a graphlet sub-orbit.

Now the goal is to enumerate all the graphlet sub-orbits. However to efficiently enumerate them, graph symmetry must be taken into consideration. A symmetry between several links exists when their attributes can be interchanged and the graphlet orbit remains the same. When no symmetry exists between links *a cross product* of the possible link attributes will exhaust all the possible configurations. Otherwise, if several links are symmetrical than taking *all the combinations with replacement* of the link attribute set, will give all the possible configurations. By using these two enumerations all the graphlet orbit forms can be distinctly identified. The composition of all the orbits for multiplex graphlets up to degree 4 are presented in Table 1.

Orbit	Orbit Alias	Orbit class		Size of orbit
0	Degree	0_{a}	$a \in E_t$	$\lvert E_t \rvert$
1	Wedge Path	$1_{[a,b]}$	$a,b \in E_t$	$\lvert E_t \rvert^2$
2	Wedge Star	$2_{\{a,b\}}$	$a,b \in E_t$	$\left(\!\binom{\lvert E_t \rvert}{2}\!\right)$
3	Triangle	$3_{[\{a,b\},c]}$	$a,b,c \in E_t$	$\left(\!\binom{\lvert E_t \rvert}{2}\!\right) \cdot \lvert E_t \rvert$
4	Four Path	$4_{[a,b,c]}$	$a,b,c \in E_t$	$\lvert E_t \rvert^3$
5		$5_{[a,[b,c]]}$	$a,b,c \in E_t$	$\lvert E_t \rvert^3$
6	Three Star	$6_{[a,\{b,c\}]}$	$a,b,c \in E_t$	$\lvert E_t \rvert \cdot \left(\!\binom{\lvert E_t \rvert}{2}\!\right)$
7		$7_{\{a,b,c\}}$	$a,b,c \in E_t$	$\left(\!\binom{\lvert E_t \rvert}{3}\!\right)$
8	Four Cycle	$8_{\{[a,d],[b,c]\}}$	$a,b,c \in E_t$	$\left(\!\binom{\lvert E_t \rvert^2}{2}\!\right)$
9	Tailed Triangle	$9_{[a,\{b,c\},d]}$	$a,b,c,d \in E_t$	$\lvert E_t \rvert \cdot \binom{\lvert E_t \rvert}{2} \cdot \lvert E_t \rvert$
10		$10_{[a,\{b,c\},d]}$	$a,b,c,d \in E_t$	$\lvert E_t \rvert \cdot \binom{\lvert E_t \rvert}{2} \cdot \lvert E_t \rvert$
11		$11_{[a,b,c,d]}$	$a,b,c,d \in E_t$	$\lvert E_t \rvert^4$
12	Chordal Cycle	$12_{[\{[a,c],[b,d]\},e]}$	$a,b,c,d \in E_t$	$\left(\!\binom{\lvert E_t \rvert^2}{2}\!\right) \cdot \lvert E_t \rvert$
13		$13_{[\{[a,b],[c,d]\},e]}$	$a,b,c,d \in E_t$	$\left(\!\binom{\lvert E_t \rvert^2}{2}\!\right) \cdot \lvert E_t \rvert$
14	Four Clique	$14_{\{a,b,c,d,e,f\}}$	$a,b,c,d,e,f \in E_t$	$\lvert E_t \rvert^5$

Table 1. Sub-orbit breakdown for graphlets up to order 4

The two possible enumerations are presented by pseudo code below. Each of them takes a list of links or link groups and enumerates them accordingly. By combining these two functions with E_t – the link attribute set, we distinctly identify all the graphlet sub-orbits up to order 3 (shown in Algorithm 1). Then once we have enumerated all the separate orbits, we can count all the degree, wedge-path wedge-star and triangle occurrences for every node in a particular graph. This is presented in Algorithm 2. [1]

[1] The code for the analysis presented in this work is located at github.

Algorithm 1 Enumeration of all the sub-orbits up to $N = 3$

```
procedure MakeMultiplexLinkConfigurations(plexes)
        ▷ Input a list of plexes, and return all the possible link configurations
    En ← []
    In ← 0
    for all n_combs ∈ 1..n do
        comb_list ← {comb|comb ∈ (plexes choose n_combs)}        ▷ Uniquely enumerate combination (n k)
        for all comb ∈ comb_list do
            En[In] ← comb
            In ← In + 1
    return En

procedure EnumerateProduct(possible_linkValMat)
        ▷ Input a list of possible link value lists, and return an indexed list of all the possible pairings
    counter ← 0
    enumerations ← []
    enum_list ← X_{i=1}^{n_needed} possible_linkValMat_i
    for all link_configuration ∈ enum_set do
        enumerations[counter] ← link_configuration
        counter ← counter + 1
    return enumerations

procedure EnumerateCombination(possible_linkVals, n_needed)
        ▷ Input a list of possible link values, and return an indexed list of all the combinations with replacement of order n
    counter ← 0
    link_length ← Length(link_list)
    enumerations ← []
    enum_set ← {comb|comb ∈ ((possible_linkVals multichoose n_needed))}        ▷ Uniquely enumerate combination with replacement ((n k))
    for all link_conf ∈ enum_set do
        enumerations[counter] ← link_conf
        counter ← counter + 1
    return enumerations

--------------------------------------------------------------------------------
procedure MakeSignatureVectorN3(plex_list)
    En ← MakeMultiplexLinkConfigurations(plex_list)
    orbit0En ← En
    orbit1En ← EnumerateProduct([En, En])
    orbit2En ← EnumerateCombination(En, 2)
    orbit3En ← EnumerateProduct([EnumerateCombination(En, 2), En])
    return [orbit0En, orbit1En, orbit2En, orbit3En]
```

Algorithm 2 Count Sub-orbits in a given graph

```
procedure RComb(N, k)
                                    ▷ count combinations (with replacement) ((n k))
    return binomial(N + k − 1, k)
procedure IterateRCombination(n, k)
        ▷ return a lexicographical ordering of a combination (with replacement) of size k, for a set with n elements
    enum_set ← {comb|comb ∈ ((n k))}      ▷ Make the set of all combinations with replacement ((n k))
    ordered_combinations ← []
    rcomb_ind ← 0
    for all rcomb_set ∈ LexicographicallyOrder(enum_set) do
        ordered_combinations[rcomb_ind] ← rcomb_set
        rcomb_ind ← rcomb_ind + 1
    return ordered_combinations
procedure CreateRCombList(N)
    ▷ Create list that returns an unique ordering of all combinations (with replacement) of size 1 − N, from a set of N elements
    rcomb_list ← []
    for all k ∈ [1..N] do
        set_size ← RComb(N, k)
        rcomb_list[end..set_size] ← IterateRCombination(N, k)
    return rcomb_list

procedure FindRCombIndex(a, b, C_E)
        ▷ calculate index of ((n k)), with a, b being the two chosen values from the set of possible link attributes C_E
    rcomb_ind ← −1
    for all comb_set ∈ CreateRCombList(C_E) do
        if comb_set then
    return IndexOfCombR_2
________________________________________________________________________
procedure CountWedgeStarsAndTriangles(V, E_A)
            ▷ calculate wedge star and triangle counts of a graph with vertices V, and edge attributes E_A
    centerWCounts ← [0...0]_v
    triCounts ← [0...0]_v
    C_E ← Unique(E_A)
    for all v ∈ V do
        for all i, j ∈ neighborhood(v) do
            edgeType_{v,i} ← E_A[v, i]
            edgeType_{v,j} ← E_A[v, j]
            if AreNeighbors(i, j) then
                edgeType_{i,j} ← E_A[i, j]
                triWedgeIndex ←
                        EnumerateCombR_2(edgeType_{v,i}, edgeType_{v,j}, C_E)
                triTailIndex ← edgeType_{i,j}
                triIndex ←
                        [triTailIndex ∗ CombR(C_E, 2)] + triWedgeIndex
                triCounts_v[triIndex] ← triCounts_v[triIndex] + 1
            else
                cWedgeIndex ←
                        EnumerateCombR_2(edgeType_{v,i}, edgeType_{v,j}, C_E)
                centerWCounts_v[cWedgeIndex] ←
                        centerWCounts_v[cWedgeIndex] + 1
    return centerWCounts, triCounts
________________________________________________________________________
```

Algorithm 2 Count Sub-orbits in a given graph (continued)

procedure COUNTWEDGEPATHS(V, E_A)
▹ calculate wedge path counts of a graph with vertices V, and edge attributes E_A
    $C_E \leftarrow Unique(E_A)$
    $pathWCounts \leftarrow [0...0]_v$
    for all $v \in V$ **do**
        for all $i \in neighborhood(v)$ **do**
            for all $j \in \{neighborhood(i) \setminus neighborhood(v)\}$ **do**
                if $j = v$ **then** Continue
                $edgeType_{v,i} \leftarrow E_A[v,i]$
                $edgeType_{i,j} \leftarrow E_A[i,j]$
                $pwedgeIndex \leftarrow [edgeType_{v,i} * C_E] + edgeType_{i,j}$
                $pathWCounts_v[pwedgeIndex] \leftarrow$
                        $pathWCounts_v[pWedgeIndex] + 1$
    return $pathWCounts$

1 Data Description

Synthetic Networks

In continuation basic description of the algorithms is provided. For each of these graph types, we then sample multiple graph instances of different sparsities and sizes. To create a multiplex network we than combine these random graphs so that each plex comes from a separate graph instance (the full sampling procedure is explained in the main text).
Watts-Strogatz – The aim of this model is to simulate a small world network. It starts with a connected n-size grid, and then a certain percentage of the grid links are interchanged, to simulate a network where the number of hops between any two nodes remains small (around 6 hops).
Erdosh Renyi – This graph model creates a graph by randomly adding edges with a probability p.
Barabashi-Albert – This model is initialized with a randomly connected network, and then in each iteration a new node is linked to an existing one. The existing node is chosen with a probability dependent on it's degree. In this way nodes with higher degrees are preferred compared to more isolated nodes.
Power-Law – These graphs are constructed in a similar manner as barabashi-albert graphs, except they use an additional triangle forming probability which randomly creates triangles instead of links.

Social Networks

In 2006 a baseline survey was conducted in a total of 75 villages. The survey consisted of a village questionnaire, a full census that collected data on all households in the villages, and a detailed follow-up survey fielded to a subsample of individuals. In the village questionnaire, data was collected regarding the village leadership, the presence of pre-existing NGOs and savings self-help groups, and various geographical features of the area (such as rivers, mountains, and roads). The household census gathered demographic information, GPS coordinates and data on a variety of amenities (such as roofing material, type of latrine, quality of access to electric power, etc.) for every household in each village. After the village and household modules were completed, a detailed individual survey was administered to a subsample of villagers. Respondents were randomly selected, and we stratified sampling by religion and geographic sub-location. The individual surveys were administered to eligible members and their spouses, yielding a sample of about 46% of all households per village. The individual questionnaire asked for information including age, sub-caste, education, language, native home, and occupation. Most importantly, these individual surveys also included a module that collected social network data along thirteen dimensions, including names of friends or relatives who visit the respondent's home, names of those friends or relatives the respondent visits, who the respondent goes to pray with (at a temple, church, or mosque), from whom the respondent would borrow money, to whom the respondent would lend money, from whom the respondent would borrow or to whom the respondent would lend material goods(kerosene, rice), from whom the respondent gets advice, and to whom the respondent gives advice. The resulting data are unusually rich, since the networks cover entire villages and 13 types of relationships between any two individuals, and since there are a large number of surveyed villages. They are publicly available on the project's web page[1].

Economy Trade Networks

To quantify the significance of export we use the Trade Intensity Index (TII). TII measures the relative advantage or disadvantage of a certain country i in the imports of country j in a certain class of products. Formally it is defined as

$$TII_{ij}^{p} = \frac{E_{ij}^{p} / \sum_j E_{ij}^{p}}{\sum_i E_{ij}^{p} / \sum_{i,j} E_{ij}^{p}},$$

where E_{ij}^{p} is the export of product p by country i in country j. A value above one implies that country i's share of export is greater than the share of overall import of product p by country j, thus indicating that the trade between the two countries is *intense*. We use this threshold to identify the links, i.e. we define the ijth entry of the adjacency matrix M^p of plex p as

$$M_{ij}^{p} = \begin{cases} 1, & \text{if } TII_{ij}^{p} \geq 1 \text{ or } TII_{ji}^{p} \geq 1 \\ 0, & \text{otherwise} \end{cases}.$$

Correlation Matrices for 2 and 3-plex networks

These are the summary correlation matrices for the two real network experiments. The first two columns in Figures 1,2 represent a summary for multiple 2-plexes (out of 100 trade classes or out of 66 relation-pairs for social 2-plexes), while the last column shows the summary for 3-plexes/4-plexes. The full procedure on the selection of 2-plexes from the full networks is described in the main text. In addition, here we display the same results for 3-plex and 4-plex.

Note that every link inside a particular sub-orbit is labeled as $(a,b,c,d)_i$ where every letter corresponds to a particular plex count $1-4$, while the index i is the distinction index of the second reduction described in the main text.

Figure 1. Summary correlation matrices from economic trade networks. We calculate strong correlations (> 0.7) of up to 2000 **individual product** (2,3,4)-plex networks, coming from 100 **product class** pairs/triplets/quadruples. For each product class tuple we extract frequent strong correlations (appearing in >60% of networks). Finally we show the number of product class tuples where a strong correlation appears. **a** show the frequency of strong 2-plex correlations with the first plex reduction (plex count), **c** shows the frequency of strong 2-plex correlations with the second plex reduction (plex-count with distinct plexes in orbit), while **b,d** show the frequent strong correlations of product class triplets/quadruples (second reduction). The colored boxes separate correlations between different orbits (0,1,2 or 3).

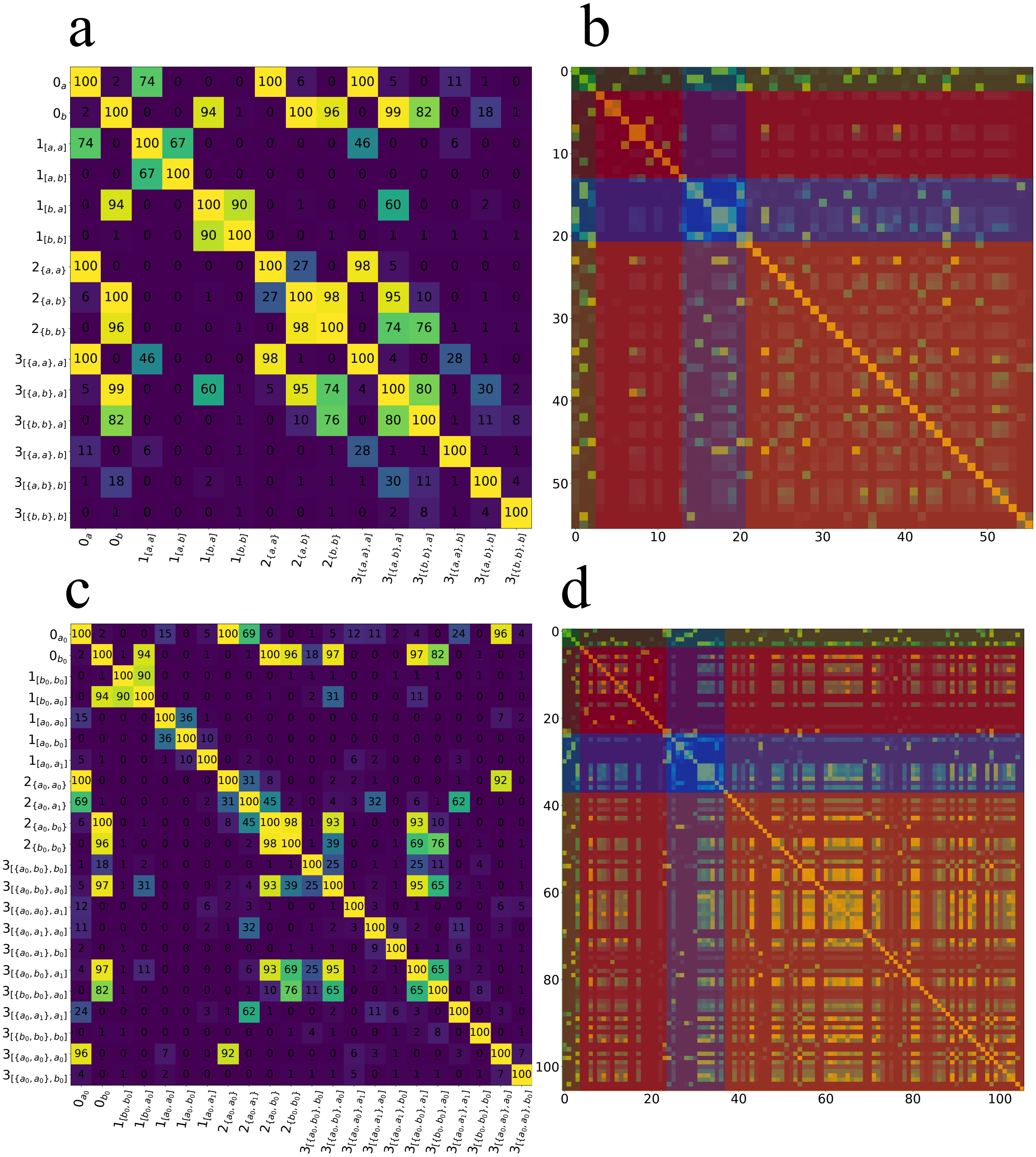

Figure 2. Summary correlation matrices from social interaction networks. We create social relation multiplex networks, where the plexes are social relations. We find strong correlations (> 0.7) in (2,3,4)-plex networks, coming from 66 or 100 social relation pairs/triplets/quadruples. For each social relation tuple we extract frequent strong correlations (appearing in >60% of villages). Finally we show the number of social relation tuples where a strong correlation appears. **a** shows the frequency of strong 2-plex correlations with the first plex reduction (plex count), **c** shows the frequency of strong 2-plex correlations with the second plex reduction (plex-count with distinct plexes in orbit), while **b,d** show the frequent strong correlations of social relation triplets/quadruples (second reduction). The colored boxes separate correlations between different orbits (0,1,2 or 3).

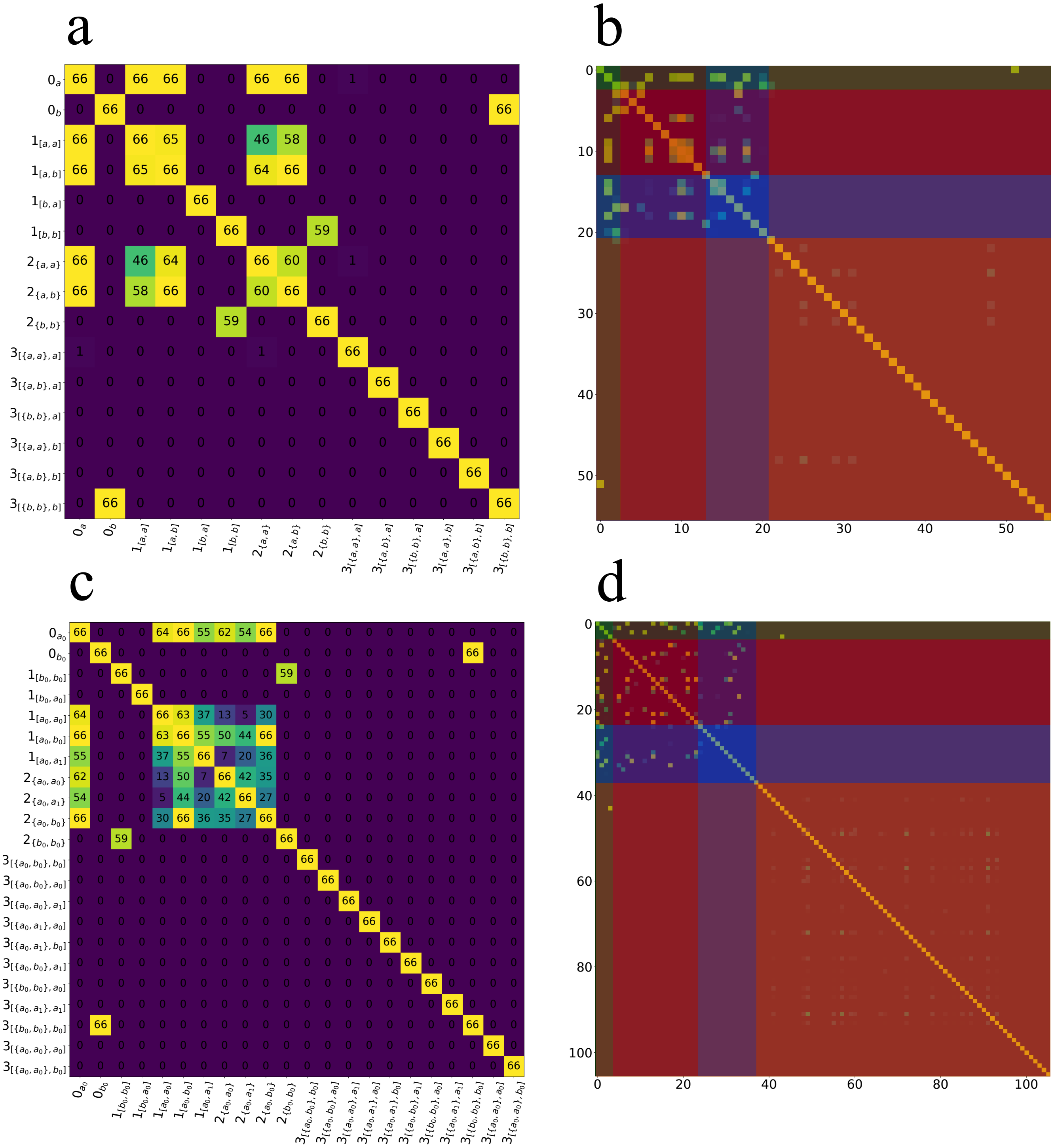

Full correlation table for Economic trade and Social relations networks

Here we present the full correlation tables (for correlations occurring more than 70% of the chosen networks) for the two real networks. We tested our method on 100 (or 66) randomly chosen 2-plexes/3-plexes/4-plexes. The procedure is thoroughly explained in the main text, with the added case of 3-plex/4-plex networks, chosen in a similar way as the 2-plexes (randomly selected product class **triplets/quadruples** or randomly chosen social relation **triplets/quadruples**). We use the second plex reduction (plex-count with distinct plexes inside orbits) in all cases.

Economic trade networks

First Sub-orbit	Second Sub-orbit	% Expressed (of product classes)
Sub-orbit: b_0, Orbit: degree	Sub-orbit: $\{a_0, b_0\}$, Orbit: wedge-star	100.00
Sub-orbit: a_0, Orbit: degree	Sub-orbit: $\{a_0, a_0\}$, Orbit: wedge-star	100.00
Sub-orbit: $\{a_0, b_0\}$, Orbit: wedge-star	Sub-orbit: $\{b_0, b_0\}$, Orbit: wedge-star	98.00
Sub-orbit: b_0, Orbit: degree	Sub-orbit: $[\{a_0, b_0\}, a_1]$, Orbit: triangle	97.00
Sub-orbit: b_0, Orbit: degree	Sub-orbit: $[\{a_0, b_0\}, a_0]$, Orbit: triangle	97.00
Sub-orbit: b_0, Orbit: degree	Sub-orbit: $\{b_0, b_0\}$, Orbit: wedge-star	96.00
Sub-orbit: a_0, Orbit: degree	Sub-orbit: $[\{a_0, a_0\}, a_0]$, Orbit: triangle	96.00
Sub-orbit: $[\{a_0, b_0\}, a_0]$, Orbit: triangle	Sub-orbit: $[\{a_0, b_0\}, a_1]$, Orbit: triangle	95.00
Sub-orbit: b_0, Orbit: degree	Sub-orbit: $[b_0, a_0]$, Orbit: wedge-path	94.00
Sub-orbit: $\{a_0, b_0\}$, Orbit: wedge-star	Sub-orbit: $[\{a_0, b_0\}, a_0]$, Orbit: triangle	93.00
Sub-orbit: $\{a_0, b_0\}$, Orbit: wedge-star	Sub-orbit: $[\{a_0, b_0\}, a_1]$, Orbit: triangle	93.00
Sub-orbit: $\{a_0, a_0\}$, Orbit: wedge-star	Sub-orbit: $[\{a_0, a_0\}, a_0]$, Orbit: triangle	92.00
Sub-orbit: $[b_0, b_0]$, Orbit: wedge-path	Sub-orbit: $[b_0, a_0]$, Orbit: wedge-path	90.00
Sub-orbit: b_0, Orbit: degree	Sub-orbit: $[\{b_0, b_0\}, a_0]$, Orbit: triangle	82.00
Sub-orbit: $\{b_0, b_0\}$, Orbit: wedge-star	Sub-orbit: $[\{b_0, b_0\}, a_0]$, Orbit: triangle	76.00

Table 2. Frequent strong correlations for 2-plex economic trade networks

First Sub-orbit	Second Sub-orbit	% Expressed (of product classes)
Sub-orbit: b_0, Orbit: degree	Sub-orbit: $[\{a_0, b_0\}, a_1]$, Orbit: triangle	100.00
Sub-orbit: b_0, Orbit: degree	Sub-orbit: $\{a_0, b_0\}$, Orbit: wedge-star	100.00
Sub-orbit: c_0, Orbit: degree	Sub-orbit: $[\{a_0, c_0\}, a_1]$, Orbit: triangle	100.00
Sub-orbit: $\{b_0, c_0\}$, Orbit: wedge-star	Sub-orbit: $\{a_0, c_0\}$, Orbit: wedge-star	100.00
Sub-orbit: a_0, Orbit: degree	Sub-orbit: $\{a_0, a_0\}$, Orbit: wedge-star	100.00
Sub-orbit: a_0, Orbit: degree	Sub-orbit: $[\{a_0, a_0\}, a_0]$, Orbit: triangle	99.00
Sub-orbit: c_0, Orbit: degree	Sub-orbit: $\{a_0, c_0\}$, Orbit: wedge-star	99.00
Sub-orbit: a_0, Orbit: degree	Sub-orbit: $\{a_0, a_1\}$, Orbit: wedge-star	99.00
Sub-orbit: b_0, Orbit: degree	Sub-orbit: $[\{a_0, b_0\}, a_0]$, Orbit: triangle	99.00
Sub-orbit: $[c_0, a_0]$, Orbit: wedge-path	Sub-orbit: $[c_0, b_0]$, Orbit: wedge-path	99.00
Sub-orbit: c_0, Orbit: degree	Sub-orbit: $[\{a_0, c_0\}, a_0]$, Orbit: triangle	98.00
Sub-orbit: c_0, Orbit: degree	Sub-orbit: $[\{b_0, c_0\}, a_0]$, Orbit: triangle	98.00
Sub-orbit: $[\{a_0, c_0\}, a_1]$, Orbit: triangle	Sub-orbit: $[\{a_0, c_0\}, a_0]$, Orbit: triangle	98.00
Sub-orbit: b_0, Orbit: degree	Sub-orbit: $\{b_0, b_0\}$, Orbit: wedge-star	98.00
Sub-orbit: $\{a_0, c_0\}$, Orbit: wedge-star	Sub-orbit: $[\{a_0, c_0\}, a_1]$, Orbit: triangle	97.00
Sub-orbit: c_0, Orbit: degree	Sub-orbit: $\{b_0, c_0\}$, Orbit: wedge-star	97.00
Sub-orbit: c_0, Orbit: degree	Sub-orbit: $[\{a_0, c_0\}, b_0]$, Orbit: triangle	96.00
Sub-orbit: $\{a_0, b_0\}$, Orbit: wedge-star	Sub-orbit: $[\{a_0, b_0\}, a_1]$, Orbit: triangle	96.00
Sub-orbit: $[\{a_0, c_0\}, a_1]$, Orbit: triangle	Sub-orbit: $[\{b_0, c_0\}, a_0]$, Orbit: triangle	96.00
Sub-orbit: $\{a_0, b_0\}$, Orbit: wedge-star	Sub-orbit: $\{b_0, b_0\}$, Orbit: wedge-star	96.00
Sub-orbit: $\{a_0, c_0\}$, Orbit: wedge-star	Sub-orbit: $[\{b_0, c_0\}, a_0]$, Orbit: triangle	94.00
Sub-orbit: $\{a_0, a_1\}$, Orbit: wedge-star	Sub-orbit: $\{a_0, b_0\}$, Orbit: wedge-star	94.00
Sub-orbit: $[\{a_0, c_0\}, a_0]$, Orbit: triangle	Sub-orbit: $[\{b_0, c_0\}, a_0]$, Orbit: triangle	93.00
Sub-orbit: $[\{a_0, c_0\}, a_1]$, Orbit: triangle	Sub-orbit: $[\{a_0, c_0\}, b_0]$, Orbit: triangle	93.00
Sub-orbit: $\{b_0, c_0\}$, Orbit: wedge-star	Sub-orbit: $[\{b_0, c_0\}, a_0]$, Orbit: triangle	93.00

Sub-orbit: $\{b_0, c_0\}$, Orbit: wedge-star	Sub-orbit: $[\{a_0, c_0\}, a_1]$, Orbit: triangle	93.00
Sub-orbit: $\{a_0, b_0\}$, Orbit: wedge-star	Sub-orbit: $[\{a_0, b_0\}, a_0]$, Orbit: triangle	93.00
Sub-orbit: $[\{a_0, b_0\}, a_0]$, Orbit: triangle	Sub-orbit: $[\{a_0, b_0\}, a_1]$, Orbit: triangle	92.00
Sub-orbit: $\{a_0, c_0\}$, Orbit: wedge-star	Sub-orbit: $[\{a_0, c_0\}, a_0]$, Orbit: triangle	92.00
Sub-orbit: c_0, Orbit: degree	Sub-orbit: $[c_0, a_0]$, Orbit: wedge-path	92.00
Sub-orbit: $[\{a_0, c_0\}, a_0]$, Orbit: triangle	Sub-orbit: $[\{a_0, c_0\}, b_0]$, Orbit: triangle	91.00
Sub-orbit: $[b_0, b_0]$, Orbit: wedge-path	Sub-orbit: $[b_0, a_0]$, Orbit: wedge-path	89.00
Sub-orbit: $[c_0, a_0]$, Orbit: wedge-path	Sub-orbit: $[\{a_0, c_0\}, b_0]$, Orbit: triangle	87.00
Sub-orbit: $\{a_0, a_0\}$, Orbit: wedge-star	Sub-orbit: $[\{a_0, a_0\}, a_0]$, Orbit: triangle	87.00
Sub-orbit: b_0, Orbit: degree	Sub-orbit: $[\{b_0, b_0\}, a_0]$, Orbit: triangle	86.00
Sub-orbit: $[a_0, a_0]$, Orbit: wedge-path	Sub-orbit: $[a_0, b_0]$, Orbit: wedge-path	86.00
Sub-orbit: b_0, Orbit: degree	Sub-orbit: $[b_0, a_0]$, Orbit: wedge-path	83.00
Sub-orbit: a_0, Orbit: degree	Sub-orbit: $[\{a_0, a_0\}, a_1]$, Orbit: triangle	82.00
Sub-orbit: a_0, Orbit: degree	Sub-orbit: $[\{a_0, a_1\}, a_1]$, Orbit: triangle	81.00
Sub-orbit: $\{a_0, a_0\}$, Orbit: wedge-star	Sub-orbit: $\{a_0, a_1\}$, Orbit: wedge-star	79.00
Sub-orbit: a_0, Orbit: degree	Sub-orbit: $[\{a_0, a_1\}, a_0]$, Orbit: triangle	79.00
Sub-orbit: $[c_0, a_0]$, Orbit: wedge-path	Sub-orbit: $[\{a_0, c_0\}, a_1]$, Orbit: triangle	79.00
Sub-orbit: $[\{b_0, c_0\}, c_0]$, Orbit: triangle	Sub-orbit: $[\{c_0, c_0\}, b_0]$, Orbit: triangle	78.00
Sub-orbit: $\{b_0, b_1\}$, Orbit: wedge-star	Sub-orbit: $[\{b_0, b_1\}, a_0]$, Orbit: triangle	78.00
Sub-orbit: $\{c_0, c_0\}$, Orbit: wedge-star	Sub-orbit: $[\{c_0, c_0\}, a_0]$, Orbit: triangle	77.00
Sub-orbit: $\{a_0, a_1\}$, Orbit: wedge-star	Sub-orbit: $[\{a_0, a_1\}, a_0]$, Orbit: triangle	77.00
Sub-orbit: $\{b_0, c_0\}$, Orbit: wedge-star	Sub-orbit: $\{c_0, c_0\}$, Orbit: wedge-star	76.00
Sub-orbit: $\{a_0, a_1\}$, Orbit: wedge-star	Sub-orbit: $[\{a_0, a_1\}, a_1]$, Orbit: triangle	76.00
Sub-orbit: $[\{b_0, c_0\}, c_0]$, Orbit: triangle	Sub-orbit: $[\{c_0, c_0\}, c_0]$, Orbit: triangle	74.00
Sub-orbit: $\{a_0, c_0\}$, Orbit: wedge-star	Sub-orbit: $\{c_0, c_0\}$, Orbit: wedge-star	74.00
Sub-orbit: $[\{c_0, c_0\}, b_0]$, Orbit: triangle	Sub-orbit: $[\{c_0, c_0\}, c_0]$, Orbit: triangle	74.00
Sub-orbit: $[a_0, b_0]$, Orbit: wedge-path	Sub-orbit: $[a_0, a_1]$, Orbit: wedge-path	73.00
Sub-orbit: $\{b_0, b_0\}$, Orbit: wedge-star	Sub-orbit: $[\{a_0, b_0\}, a_1]$, Orbit: triangle	71.00
Sub-orbit: $\{a_0, a_0\}$, Orbit: wedge-star	Sub-orbit: $\{a_0, b_0\}$, Orbit: wedge-star	71.00

Table 3. Frequent strong correlations for 3-plex economic trade networks

First Sub-orbit	Second Sub-orbit	% Expressed (of product classes)
Sub-orbit: $\{a_0, b_0\}$, Orbit: wedge-star	Sub-orbit: $[\{a_0, b_0\}, a_1]$, Orbit: triangle	100.00
Sub-orbit: $[\{a_0, d_0\}, b_0]$, Orbit: triangle	Sub-orbit: $[\{b_0, d_0\}, b_1]$, Orbit: triangle	100.00
Sub-orbit: $[\{a_0, d_0\}, b_0]$, Orbit: triangle	Sub-orbit: $[\{a_0, d_0\}, a_0]$, Orbit: triangle	100.00
Sub-orbit: $[\{a_0, d_0\}, b_0]$, Orbit: triangle	Sub-orbit: $[\{a_0, d_0\}, a_1]$, Orbit: triangle	100.00
Sub-orbit: $\{a_0, d_0\}$, Orbit: wedge-star	Sub-orbit: $[\{a_0, d_0\}, a_1]$, Orbit: triangle	100.00
Sub-orbit: $[\{d_0, d_0\}, d_0]$, Orbit: triangle	Sub-orbit: $[\{d_0, d_0\}, c_0]$, Orbit: triangle	100.00
Sub-orbit: c_0, Orbit: degree	Sub-orbit: $[\{a_0, c_0\}, a_1]$, Orbit: triangle	100.00
Sub-orbit: c_0, Orbit: degree	Sub-orbit: $[\{a_0, c_0\}, a_0]$, Orbit: triangle	100.00
Sub-orbit: $[\{c_0, d_0\}, a_0]$, Orbit: triangle	Sub-orbit: $[\{a_0, d_0\}, a_1]$, Orbit: triangle	100.00
Sub-orbit: $[\{b_0, d_0\}, b_1]$, Orbit: triangle	Sub-orbit: $[\{a_0, d_0\}, a_0]$, Orbit: triangle	100.00
Sub-orbit: c_0, Orbit: degree	Sub-orbit: $[\{b_0, c_0\}, a_0]$, Orbit: triangle	100.00
Sub-orbit: $[\{b_0, d_0\}, b_1]$, Orbit: triangle	Sub-orbit: $[\{a_0, d_0\}, a_1]$, Orbit: triangle	100.00
Sub-orbit: c_0, Orbit: degree	Sub-orbit: $\{a_0, c_0\}$, Orbit: wedge-star	100.00
Sub-orbit: $[\{c_0, d_0\}, c_0]$, Orbit: triangle	Sub-orbit: $[\{c_0, c_0\}, d_0]$, Orbit: triangle	100.00
Sub-orbit: $\{c_0, d_0\}$, Orbit: wedge-star	Sub-orbit: $\{b_0, d_0\}$, Orbit: wedge-star	100.00
Sub-orbit: $[\{c_0, d_0\}, d_0]$, Orbit: triangle	Sub-orbit: $[\{d_0, d_0\}, c_0]$, Orbit: triangle	100.00
Sub-orbit: b_0, Orbit: degree	Sub-orbit: $[\{a_0, b_0\}, a_1]$, Orbit: triangle	100.00
Sub-orbit: $\{b_0, c_0\}$, Orbit: wedge-star	Sub-orbit: $\{a_0, c_0\}$, Orbit: wedge-star	100.00
Sub-orbit: $[\{a_0, b_0\}, a_0]$, Orbit: triangle	Sub-orbit: $[\{a_0, b_0\}, a_1]$, Orbit: triangle	100.00
Sub-orbit: $[\{a_0, d_0\}, a_0]$, Orbit: triangle	Sub-orbit: $[\{a_0, d_0\}, a_1]$, Orbit: triangle	100.00
Sub-orbit: $\{c_0, d_0\}$, Orbit: wedge-star	Sub-orbit: $[\{b_0, d_0\}, a_0]$, Orbit: triangle	100.00
Sub-orbit: b_0, Orbit: degree	Sub-orbit: $[\{a_0, b_0\}, a_0]$, Orbit: triangle	100.00

Sub-orbit: $\{a_0,c_0\}$, Orbit: wedge-star	Sub-orbit: $[\{a_0,c_0\},a_1]$, Orbit: triangle	100.00
Sub-orbit: a_0, Orbit: degree	Sub-orbit: $[\{a_0,a_0\},a_0]$, Orbit: triangle	100.00
Sub-orbit: b_0, Orbit: degree	Sub-orbit: $\{b_0,b_0\}$, Orbit: wedge-star	100.00
Sub-orbit: b_0, Orbit: degree	Sub-orbit: $\{a_0,b_0\}$, Orbit: wedge-star	100.00
Sub-orbit: a_0, Orbit: degree	Sub-orbit: $\{a_0,a_0\}$, Orbit: wedge-star	100.00
Sub-orbit: $\{b_0,d_0\}$, Orbit: wedge-star	Sub-orbit: $[\{b_0,d_0\},a_0]$, Orbit: triangle	100.00
Sub-orbit: $[\{a_0,d_0\},c_0]$, Orbit: triangle	Sub-orbit: $[\{a_0,d_0\},b_0]$, Orbit: triangle	100.00
Sub-orbit: $[\{b_0,d_0\},a_0]$, Orbit: triangle	Sub-orbit: $[\{a_0,d_0\},b_0]$, Orbit: triangle	100.00
Sub-orbit: $[\{b_0,d_0\},a_0]$, Orbit: triangle	Sub-orbit: $[\{c_0,d_0\},a_0]$, Orbit: triangle	100.00
Sub-orbit: d_0, Orbit: degree	Sub-orbit: $[\{a_0,d_0\},a_0]$, Orbit: triangle	100.00
Sub-orbit: $\{a_0,d_0\}$, Orbit: wedge-star	Sub-orbit: $\{b_0,d_0\}$, Orbit: wedge-star	100.00
Sub-orbit: d_0, Orbit: degree	Sub-orbit: $\{a_0,d_0\}$, Orbit: wedge-star	100.00
Sub-orbit: $[\{b_0,d_0\},a_0]$, Orbit: triangle	Sub-orbit: $[\{a_0,d_0\},a_0]$, Orbit: triangle	100.00
Sub-orbit: $[\{b_0,d_0\},a_0]$, Orbit: triangle	Sub-orbit: $[\{a_0,d_0\},a_1]$, Orbit: triangle	100.00
Sub-orbit: d_0, Orbit: degree	Sub-orbit: $[\{a_0,d_0\},a_1]$, Orbit: triangle	100.00
Sub-orbit: d_0, Orbit: degree	Sub-orbit: $[\{b_0,d_0\},b_1]$, Orbit: triangle	100.00
Sub-orbit: $[\{b_0,d_0\},a_0]$, Orbit: triangle	Sub-orbit: $[\{b_0,d_0\},b_1]$, Orbit: triangle	100.00
Sub-orbit: $[c_0,a_0]$, Orbit: wedge-path	Sub-orbit: $[c_0,b_0]$, Orbit: wedge-path	100.00
Sub-orbit: $[\{d_0,d_0\},d_0]$, Orbit: triangle	Sub-orbit: $[\{c_0,d_0\},d_0]$, Orbit: triangle	100.00
Sub-orbit: d_0, Orbit: degree	Sub-orbit: $[\{a_0,d_0\},b_0]$, Orbit: triangle	100.00
Sub-orbit: $[\{d_0,d_0\},d_0]$, Orbit: triangle	Sub-orbit: $[\{c_0,d_0\},c_0]$, Orbit: triangle	100.00
Sub-orbit: $[d_0,a_0]$, Orbit: wedge-path	Sub-orbit: $[d_0,b_0]$, Orbit: wedge-path	100.00
Sub-orbit: $\{a_0,b_0\}$, Orbit: wedge-star	Sub-orbit: $\{b_0,b_0\}$, Orbit: wedge-star	100.00
Sub-orbit: d_0, Orbit: degree	Sub-orbit: $\{b_0,d_0\}$, Orbit: wedge-star	100.00
Sub-orbit: $[d_0,c_0]$, Orbit: wedge-path	Sub-orbit: $[d_0,b_0]$, Orbit: wedge-path	100.00
Sub-orbit: $[\{d_0,d_0\},d_0]$, Orbit: triangle	Sub-orbit: $[\{c_0,c_0\},d_0]$, Orbit: triangle	100.00
Sub-orbit: a_0, Orbit: degree	Sub-orbit: $\{a_0,a_1\}$, Orbit: wedge-star	100.00
Sub-orbit: $[d_0,a_0]$, Orbit: wedge-path	Sub-orbit: $[\{a_0,d_0\},b_0]$, Orbit: triangle	100.00
Sub-orbit: $\{a_0,d_0\}$, Orbit: wedge-star	Sub-orbit: $[\{a_0,d_0\},a_0]$, Orbit: triangle	100.00
Sub-orbit: d_0, Orbit: degree	Sub-orbit: $[\{b_0,d_0\},a_0]$, Orbit: triangle	100.00
Sub-orbit: $\{a_0,d_0\}$, Orbit: wedge-star	Sub-orbit: $[\{b_0,d_0\},a_0]$, Orbit: triangle	100.00
Sub-orbit: $[\{d_0,d_0\},b_0]$, Orbit: triangle	Sub-orbit: $[\{d_0,d_0\},c_0]$, Orbit: triangle	99.00
Sub-orbit: $[\{b_0,d_0\},d_0]$, Orbit: triangle	Sub-orbit: $[\{d_0,d_0\},b_0]$, Orbit: triangle	99.00
Sub-orbit: $[\{c_0,c_1\},c_1]$, Orbit: triangle	Sub-orbit: $[\{d_0,d_0\},c_0]$, Orbit: triangle	99.00
Sub-orbit: $[\{b_0,d_0\},d_0]$, Orbit: triangle	Sub-orbit: $[\{d_0,d_0\},c_0]$, Orbit: triangle	99.00
Sub-orbit: $[\{c_0,d_0\},d_0]$, Orbit: triangle	Sub-orbit: $[\{c_0,d_0\},c_0]$, Orbit: triangle	99.00
Sub-orbit: $[d_0,a_0]$, Orbit: wedge-path	Sub-orbit: $[d_0,c_0]$, Orbit: wedge-path	99.00
Sub-orbit: d_0, Orbit: degree	Sub-orbit: $\{c_0,d_0\}$, Orbit: wedge-star	99.00
Sub-orbit: $[\{c_0,d_0\},c_0]$, Orbit: triangle	Sub-orbit: $[\{b_0,d_0\},d_0]$, Orbit: triangle	99.00
Sub-orbit: $[\{c_0,d_0\},d_0]$, Orbit: triangle	Sub-orbit: $[\{c_0,c_0\},d_0]$, Orbit: triangle	99.00
Sub-orbit: $[\{c_0,d_0\},d_0]$, Orbit: triangle	Sub-orbit: $[\{d_0,d_0\},b_0]$, Orbit: triangle	99.00
Sub-orbit: $\{c_0,d_0\}$, Orbit: wedge-star	Sub-orbit: $\{d_0,d_0\}$, Orbit: wedge-star	99.00
Sub-orbit: $\{b_0,d_0\}$, Orbit: wedge-star	Sub-orbit: $[\{a_0,d_0\},a_1]$, Orbit: triangle	99.00
Sub-orbit: $\{a_0,d_0\}$, Orbit: wedge-star	Sub-orbit: $\{c_0,d_0\}$, Orbit: wedge-star	99.00
Sub-orbit: $[\{c_0,d_0\},c_0]$, Orbit: triangle	Sub-orbit: $[\{d_0,d_0\},b_0]$, Orbit: triangle	99.00
Sub-orbit: $[\{c_0,d_0\},c_0]$, Orbit: triangle	Sub-orbit: $[\{d_0,d_0\},c_0]$, Orbit: triangle	99.00
Sub-orbit: $[\{d_0,d_0\},a_0]$, Orbit: triangle	Sub-orbit: $[\{d_0,d_0\},b_0]$, Orbit: triangle	99.00
Sub-orbit: $[\{c_0,c_0\},d_0]$, Orbit: triangle	Sub-orbit: $[\{d_0,d_0\},b_0]$, Orbit: triangle	99.00
Sub-orbit: $[\{c_0,c_1\},c_1]$, Orbit: triangle	Sub-orbit: $[\{c_0,d_0\},d_0]$, Orbit: triangle	99.00
Sub-orbit: d_0, Orbit: degree	Sub-orbit: $[d_0,a_0]$, Orbit: wedge-path	99.00
Sub-orbit: $[\{c_0,c_0\},d_0]$, Orbit: triangle	Sub-orbit: $[\{d_0,d_0\},c_0]$, Orbit: triangle	99.00
Sub-orbit: $\{a_0,d_0\}$, Orbit: wedge-star	Sub-orbit: $[\{c_0,d_0\},a_0]$, Orbit: triangle	99.00
Sub-orbit: $[\{c_0,d_0\},d_0]$, Orbit: triangle	Sub-orbit: $[\{b_0,d_0\},d_0]$, Orbit: triangle	99.00
Sub-orbit: $[\{c_0,d_0\},c_1]$, Orbit: triangle	Sub-orbit: $[\{d_0,d_0\},b_0]$, Orbit: triangle	99.00
Sub-orbit: c_0, Orbit: degree	Sub-orbit: $\{b_0,c_0\}$, Orbit: wedge-star	99.00

Sub-orbit: $[\{b_0,d_0\},d_0]$, Orbit: triangle	Sub-orbit: $[\{c_0,c_0\},d_0]$, Orbit: triangle	99.00
Sub-orbit: $[\{d_0,d_0\},d_0]$, Orbit: triangle	Sub-orbit: $[\{b_0,d_0\},d_0]$, Orbit: triangle	99.00
Sub-orbit: $[\{d_0,d_0\},d_0]$, Orbit: triangle	Sub-orbit: $[\{c_0,c_1\},c_1]$, Orbit: triangle	99.00
Sub-orbit: $[\{d_0,d_0\},d_0]$, Orbit: triangle	Sub-orbit: $[\{c_0,c_1\},c_0]$, Orbit: triangle	99.00
Sub-orbit: $\{a_0,a_1\}$, Orbit: wedge-star	Sub-orbit: $\{a_0,b_0\}$, Orbit: wedge-star	99.00
Sub-orbit: $\{a_0,b_0\}$, Orbit: wedge-star	Sub-orbit: $[\{a_0,b_0\},a_0]$, Orbit: triangle	99.00
Sub-orbit: $[\{d_0,d_0\},d_0]$, Orbit: triangle	Sub-orbit: $[\{d_0,d_0\},b_0]$, Orbit: triangle	99.00
Sub-orbit: $[\{d_0,d_0\},d_0]$, Orbit: triangle	Sub-orbit: $[\{c_0,c_0\},c_0]$, Orbit: triangle	99.00
Sub-orbit: $[\{c_0,c_1\},d_0]$, Orbit: triangle	Sub-orbit: $[\{d_0,d_0\},b_0]$, Orbit: triangle	98.00
Sub-orbit: $\{a_0,c_0\}$, Orbit: wedge-star	Sub-orbit: $[\{b_0,c_0\},a_0]$, Orbit: triangle	98.00
Sub-orbit: $[\{c_0,c_1\},d_0]$, Orbit: triangle	Sub-orbit: $[\{b_0,d_0\},d_0]$, Orbit: triangle	98.00
Sub-orbit: $[\{c_0,c_1\},d_0]$, Orbit: triangle	Sub-orbit: $[\{c_0,c_0\},d_0]$, Orbit: triangle	98.00
Sub-orbit: $[\{d_0,d_0\},d_0]$, Orbit: triangle	Sub-orbit: $[\{c_0,d_0\},c_1]$, Orbit: triangle	98.00
Sub-orbit: $[\{a_0,c_0\},a_0]$, Orbit: triangle	Sub-orbit: $[\{a_0,c_0\},a_1]$, Orbit: triangle	98.00
Sub-orbit: c_0, Orbit: degree	Sub-orbit: $[\{a_0,c_0\},b_0]$, Orbit: triangle	98.00
Sub-orbit: $[\{d_0,d_0\},d_0]$, Orbit: triangle	Sub-orbit: $[\{c_0,c_1\},c_2]$, Orbit: triangle	98.00
Sub-orbit: $[\{c_0,c_1\},d_0]$, Orbit: triangle	Sub-orbit: $[\{d_0,d_0\},c_0]$, Orbit: triangle	98.00
Sub-orbit: $[\{c_0,d_0\},d_0]$, Orbit: triangle	Sub-orbit: $[\{c_0,c_1\},c_0]$, Orbit: triangle	98.00
Sub-orbit: $[\{c_0,d_0\},d_0]$, Orbit: triangle	Sub-orbit: $[\{c_0,c_1\},d_0]$, Orbit: triangle	98.00
Sub-orbit: d_0, Orbit: degree	Sub-orbit: $[\{c_0,d_0\},a_0]$, Orbit: triangle	98.00
Sub-orbit: $\{d_0,d_0\}$, Orbit: wedge-star	Sub-orbit: $[\{d_0,d_0\},b_0]$, Orbit: triangle	98.00
Sub-orbit: $[\{b_0,d_0\},b_1]$, Orbit: triangle	Sub-orbit: $[\{c_0,d_0\},a_0]$, Orbit: triangle	98.00
Sub-orbit: $[\{c_0,d_0\},b_0]$, Orbit: triangle	Sub-orbit: $[\{c_0,d_0\},a_0]$, Orbit: triangle	98.00
Sub-orbit: $[\{c_0,c_1\},c_0]$, Orbit: triangle	Sub-orbit: $[\{d_0,d_0\},c_0]$, Orbit: triangle	98.00
Sub-orbit: $[\{c_0,d_0\},c_0]$, Orbit: triangle	Sub-orbit: $[\{c_0,c_1\},d_0]$, Orbit: triangle	98.00
Sub-orbit: $\{b_0,d_0\}$, Orbit: wedge-star	Sub-orbit: $[\{c_0,d_0\},a_0]$, Orbit: triangle	98.00
Sub-orbit: $[\{c_0,d_0\},c_0]$, Orbit: triangle	Sub-orbit: $[\{a_0,d_0\},d_0]$, Orbit: triangle	98.00
Sub-orbit: $[\{d_0,d_0\},d_0]$, Orbit: triangle	Sub-orbit: $[\{a_0,d_0\},d_0]$, Orbit: triangle	98.00
Sub-orbit: $[\{c_0,d_0\},c_0]$, Orbit: triangle	Sub-orbit: $[\{d_0,d_0\},a_0]$, Orbit: triangle	98.00
Sub-orbit: $[\{c_0,c_0\},d_0]$, Orbit: triangle	Sub-orbit: $[\{d_0,d_0\},a_0]$, Orbit: triangle	98.00
Sub-orbit: $\{d_0,d_0\}$, Orbit: wedge-star	Sub-orbit: $[\{d_0,d_0\},a_0]$, Orbit: triangle	98.00
Sub-orbit: $[\{d_0,d_0\},a_0]$, Orbit: triangle	Sub-orbit: $[\{d_0,d_0\},c_0]$, Orbit: triangle	98.00
Sub-orbit: $[\{d_0,d_0\},d_0]$, Orbit: triangle	Sub-orbit: $[\{c_0,c_1\},d_0]$, Orbit: triangle	98.00
Sub-orbit: $[\{d_0,d_0\},d_0]$, Orbit: triangle	Sub-orbit: $[\{d_0,d_0\},a_0]$, Orbit: triangle	98.00
Sub-orbit: $[\{c_0,d_0\},c_1]$, Orbit: triangle	Sub-orbit: $[\{c_0,d_0\},d_0]$, Orbit: triangle	98.00
Sub-orbit: $[\{c_0,d_0\},c_1]$, Orbit: triangle	Sub-orbit: $[\{c_0,d_0\},c_0]$, Orbit: triangle	98.00
Sub-orbit: $[\{c_0,d_0\},c_1]$, Orbit: triangle	Sub-orbit: $[\{d_0,d_0\},c_0]$, Orbit: triangle	98.00
Sub-orbit: $[\{c_0,d_0\},c_1]$, Orbit: triangle	Sub-orbit: $[\{b_0,d_0\},d_0]$, Orbit: triangle	98.00
Sub-orbit: $[\{a_0,d_0\},d_0]$, Orbit: triangle	Sub-orbit: $[\{d_0,d_0\},a_0]$, Orbit: triangle	98.00
Sub-orbit: $[\{a_0,d_0\},d_0]$, Orbit: triangle	Sub-orbit: $[\{c_0,c_0\},d_0]$, Orbit: triangle	98.00
Sub-orbit: $[\{c_0,d_0\},c_1]$, Orbit: triangle	Sub-orbit: $[\{c_0,c_0\},d_0]$, Orbit: triangle	98.00
Sub-orbit: $[\{c_0,d_0\},c_1]$, Orbit: triangle	Sub-orbit: $[\{c_0,c_1\},d_0]$, Orbit: triangle	98.00
Sub-orbit: $[\{c_0,c_0\},d_0]$, Orbit: triangle	Sub-orbit: $[\{c_0,c_1\},c_2]$, Orbit: triangle	97.00
Sub-orbit: $[\{c_0,d_0\},c_1]$, Orbit: triangle	Sub-orbit: $[\{d_0,d_0\},a_0]$, Orbit: triangle	97.00
Sub-orbit: $[\{a_0,d_0\},d_0]$, Orbit: triangle	Sub-orbit: $[\{b_0,d_0\},d_0]$, Orbit: triangle	97.00
Sub-orbit: $[\{a_0,d_0\},d_0]$, Orbit: triangle	Sub-orbit: $[\{d_0,d_0\},b_0]$, Orbit: triangle	97.00
Sub-orbit: $[\{b_0,d_0\},a_0]$, Orbit: triangle	Sub-orbit: $[\{c_0,d_0\},b_0]$, Orbit: triangle	97.00
Sub-orbit: $[\{c_0,d_0\},d_0]$, Orbit: triangle	Sub-orbit: $[\{c_0,c_1\},c_2]$, Orbit: triangle	97.00
Sub-orbit: $\{c_0,d_0\}$, Orbit: wedge-star	Sub-orbit: $[\{c_0,d_0\},a_0]$, Orbit: triangle	97.00
Sub-orbit: $[\{c_0,d_0\},d_0]$, Orbit: triangle	Sub-orbit: $[\{d_0,d_0\},a_0]$, Orbit: triangle	97.00
Sub-orbit: a_0, Orbit: degree	Sub-orbit: $[\{a_0,a_1\},a_0]$, Orbit: triangle	97.00
Sub-orbit: $[\{b_0,c_0\},a_0]$, Orbit: triangle	Sub-orbit: $[\{a_0,c_0\},a_1]$, Orbit: triangle	97.00
Sub-orbit: $[\{a_0,d_0\},d_0]$, Orbit: triangle	Sub-orbit: $[\{d_0,d_0\},c_0]$, Orbit: triangle	97.00
Sub-orbit: $[\{c_0,d_0\},c_0]$, Orbit: triangle	Sub-orbit: $[\{c_0,c_1\},c_0]$, Orbit: triangle	97.00
Sub-orbit: $[\{b_0,d_0\},d_0]$, Orbit: triangle	Sub-orbit: $[\{d_0,d_0\},a_0]$, Orbit: triangle	97.00

Sub-orbit: $[\{c_0,d_0\},c_0]$, Orbit: triangle	Sub-orbit: $[\{c_0,c_1\},c_2]$, Orbit: triangle	97.00
Sub-orbit: $\{b_0,d_0\}$, Orbit: wedge-star	Sub-orbit: $[\{a_0,d_0\},a_0]$, Orbit: triangle	97.00
Sub-orbit: $[\{c_0,c_1\},c_0]$, Orbit: triangle	Sub-orbit: $[\{c_0,c_1\},c_2]$, Orbit: triangle	97.00
Sub-orbit: $[\{d_0,d_0\},b_0]$, Orbit: triangle	Sub-orbit: $[\{c_0,c_1\},c_2]$, Orbit: triangle	97.00
Sub-orbit: $[\{b_0,d_0\},b_1]$, Orbit: triangle	Sub-orbit: $[\{c_0,d_0\},b_0]$, Orbit: triangle	97.00
Sub-orbit: $\{d_0,d_0\}$, Orbit: wedge-star	Sub-orbit: $[\{d_0,d_0\},c_0]$, Orbit: triangle	97.00
Sub-orbit: $[\{c_0,c_1\},c_0]$, Orbit: triangle	Sub-orbit: $[\{c_0,c_1\},d_0]$, Orbit: triangle	97.00
Sub-orbit: $[\{c_0,c_1\},c_0]$, Orbit: triangle	Sub-orbit: $[\{d_0,d_0\},b_0]$, Orbit: triangle	97.00
Sub-orbit: a_0, Orbit: degree	Sub-orbit: $[\{a_0,a_0\},a_1]$, Orbit: triangle	97.00
Sub-orbit: $[d_0,b_0]$, Orbit: wedge-path	Sub-orbit: $[\{a_0,d_0\},b_0]$, Orbit: triangle	97.00
Sub-orbit: $[\{c_0,c_1\},c_0]$, Orbit: triangle	Sub-orbit: $[\{b_0,d_0\},d_0]$, Orbit: triangle	97.00
Sub-orbit: $[\{c_0,c_1\},c_0]$, Orbit: triangle	Sub-orbit: $[\{c_0,c_0\},d_0]$, Orbit: triangle	97.00
Sub-orbit: $[\{c_0,c_1\},c_2]$, Orbit: triangle	Sub-orbit: $[\{d_0,d_0\},c_0]$, Orbit: triangle	97.00
Sub-orbit: $[\{c_0,d_0\},d_0]$, Orbit: triangle	Sub-orbit: $[\{a_0,d_0\},d_0]$, Orbit: triangle	97.00
Sub-orbit: $\{b_0,c_0\}$, Orbit: wedge-star	Sub-orbit: $[\{b_0,c_0\},a_0]$, Orbit: triangle	96.00
Sub-orbit: $[\{b_0,d_0\},d_0]$, Orbit: triangle	Sub-orbit: $[\{c_0,c_1\},c_2]$, Orbit: triangle	96.00
Sub-orbit: $[d_0,d_0]$, Orbit: wedge-path	Sub-orbit: $[\{d_0,d_0\},c_0]$, Orbit: triangle	96.00
Sub-orbit: $[\{c_0,d_0\},d_0]$, Orbit: triangle	Sub-orbit: $[\{c_0,c_0\},c_0]$, Orbit: triangle	96.00
Sub-orbit: $[\{c_0,c_0\},c_0]$, Orbit: triangle	Sub-orbit: $[\{d_0,d_0\},c_0]$, Orbit: triangle	96.00
Sub-orbit: $[d_0,d_0]$, Orbit: wedge-path	Sub-orbit: $\{d_0,d_0\}$, Orbit: wedge-star	96.00
Sub-orbit: a_0, Orbit: degree	Sub-orbit: $[\{a_0,a_1\},a_1]$, Orbit: triangle	96.00
Sub-orbit: $[d_0,a_0]$, Orbit: wedge-path	Sub-orbit: $[\{a_0,d_0\},c_0]$, Orbit: triangle	96.00
Sub-orbit: $\{d_0,d_0\}$, Orbit: wedge-star	Sub-orbit: $[\{c_0,d_0\},d_0]$, Orbit: triangle	96.00
Sub-orbit: $\{d_0,d_0\}$, Orbit: wedge-star	Sub-orbit: $[\{d_0,d_0\},d_0]$, Orbit: triangle	96.00
Sub-orbit: $[\{b_0,d_0\},b_1]$, Orbit: triangle	Sub-orbit: $[\{b_0,d_0\},c_0]$, Orbit: triangle	96.00
Sub-orbit: $[\{c_0,d_0\},c_1]$, Orbit: triangle	Sub-orbit: $[\{c_0,c_1\},c_0]$, Orbit: triangle	96.00
Sub-orbit: $[d_0,b_0]$, Orbit: wedge-path	Sub-orbit: $[\{a_0,d_0\},c_0]$, Orbit: triangle	96.00
Sub-orbit: $\{d_0,d_0\}$, Orbit: wedge-star	Sub-orbit: $[\{c_0,d_0\},c_0]$, Orbit: triangle	96.00
Sub-orbit: $[\{c_0,c_1\},c_1]$, Orbit: triangle	Sub-orbit: $[\{c_0,d_0\},c_0]$, Orbit: triangle	96.00
Sub-orbit: $\{a_0,d_0\}$, Orbit: wedge-star	Sub-orbit: $[\{b_0,d_0\},b_1]$, Orbit: triangle	96.00
Sub-orbit: $[d_0,d_0]$, Orbit: wedge-path	Sub-orbit: $[\{c_0,d_0\},d_0]$, Orbit: triangle	96.00
Sub-orbit: $[\{c_0,c_1\},c_1]$, Orbit: triangle	Sub-orbit: $[\{c_0,c_0\},d_0]$, Orbit: triangle	96.00
Sub-orbit: $[d_0,a_0]$, Orbit: wedge-path	Sub-orbit: $[\{a_0,d_0\},a_1]$, Orbit: triangle	96.00
Sub-orbit: $\{d_0,d_0\}$, Orbit: wedge-star	Sub-orbit: $[\{b_0,d_0\},d_0]$, Orbit: triangle	96.00
Sub-orbit: $[\{c_0,d_0\},b_0]$, Orbit: triangle	Sub-orbit: $[\{a_0,d_0\},a_1]$, Orbit: triangle	96.00
Sub-orbit: a_0, Orbit: degree	Sub-orbit: $\{a_0,b_0\}$, Orbit: wedge-star	96.00
Sub-orbit: $[d_0,d_0]$, Orbit: wedge-path	Sub-orbit: $[\{d_0,d_0\},d_0]$, Orbit: triangle	96.00
Sub-orbit: $[\{a_0,d_0\},a_0]$, Orbit: triangle	Sub-orbit: $[\{c_0,d_0\},a_0]$, Orbit: triangle	96.00
Sub-orbit: $[\{c_0,c_1\},c_1]$, Orbit: triangle	Sub-orbit: $[\{b_0,d_0\},d_0]$, Orbit: triangle	95.00
Sub-orbit: $[\{d_0,d_0\},d_0]$, Orbit: triangle	Sub-orbit: $[\{c_0,c_0\},c_1]$, Orbit: triangle	95.00
Sub-orbit: $[\{c_0,c_0\},c_1]$, Orbit: triangle	Sub-orbit: $[\{d_0,d_0\},c_0]$, Orbit: triangle	95.00
Sub-orbit: $[\{c_0,c_1\},c_0]$, Orbit: triangle	Sub-orbit: $[\{a_0,d_0\},d_0]$, Orbit: triangle	95.00
Sub-orbit: $\{d_0,d_0\}$, Orbit: wedge-star	Sub-orbit: $[\{c_0,c_0\},d_0]$, Orbit: triangle	95.00
Sub-orbit: $[\{c_0,c_1\},c_1]$, Orbit: triangle	Sub-orbit: $[\{c_0,c_0\},c_1]$, Orbit: triangle	95.00
Sub-orbit: $[\{c_0,d_0\},c_1]$, Orbit: triangle	Sub-orbit: $[\{c_0,c_1\},c_2]$, Orbit: triangle	95.00
Sub-orbit: $[\{c_0,c_1\},c_1]$, Orbit: triangle	Sub-orbit: $[\{d_0,d_0\},a_0]$, Orbit: triangle	95.00
Sub-orbit: $[\{c_0,c_1\},c_1]$, Orbit: triangle	Sub-orbit: $[\{c_0,c_1\},c_0]$, Orbit: triangle	95.00
Sub-orbit: $[\{c_0,c_1\},c_1]$, Orbit: triangle	Sub-orbit: $[\{c_0,c_1\},d_0]$, Orbit: triangle	95.00
Sub-orbit: $[\{c_0,c_1\},c_1]$, Orbit: triangle	Sub-orbit: $[\{d_0,d_0\},b_0]$, Orbit: triangle	95.00
Sub-orbit: $[\{c_0,c_1\},d_0]$, Orbit: triangle	Sub-orbit: $[\{c_0,c_1\},c_2]$, Orbit: triangle	95.00
Sub-orbit: $[\{c_0,d_0\},c_0]$, Orbit: triangle	Sub-orbit: $[\{c_0,c_0\},c_0]$, Orbit: triangle	95.00
Sub-orbit: $[\{b_0,d_0\},b_0]$, Orbit: triangle	Sub-orbit: $[\{d_0,d_0\},c_0]$, Orbit: triangle	95.00
Sub-orbit: d_0, Orbit: degree	Sub-orbit: $[\{a_0,d_0\},c_0]$, Orbit: triangle	95.00
Sub-orbit: $\{d_0,d_0\}$, Orbit: wedge-star	Sub-orbit: $[\{c_0,d_0\},c_1]$, Orbit: triangle	95.00
Sub-orbit: $[\{c_0,d_0\},c_1]$, Orbit: triangle	Sub-orbit: $[\{a_0,d_0\},d_0]$, Orbit: triangle	95.00

Sub-orbit: $[\{c_0,c_0\},c_0]$, Orbit: triangle	Sub-orbit: $[\{c_0,c_0\},d_0]$, Orbit: triangle	95.00
Sub-orbit: $[\{c_0,c_1\},c_1]$, Orbit: triangle	Sub-orbit: $[\{c_0,c_1\},c_2]$, Orbit: triangle	95.00
Sub-orbit: $[\{c_0,d_0\},c_1]$, Orbit: triangle	Sub-orbit: $[\{c_0,c_1\},c_1]$, Orbit: triangle	95.00
Sub-orbit: $[\{d_0,d_0\},a_0]$, Orbit: triangle	Sub-orbit: $[\{c_0,c_1\},c_2]$, Orbit: triangle	95.00
Sub-orbit: $[\{c_0,d_0\},d_0]$, Orbit: triangle	Sub-orbit: $[\{c_0,c_0\},c_1]$, Orbit: triangle	95.00
Sub-orbit: $[\{a_0,d_0\},d_0]$, Orbit: triangle	Sub-orbit: $[\{c_0,c_1\},c_2]$, Orbit: triangle	95.00
Sub-orbit: $[\{a_0,d_0\},c_0]$, Orbit: triangle	Sub-orbit: $[\{a_0,d_0\},a_1]$, Orbit: triangle	95.00
Sub-orbit: $[d_0,d_0]$, Orbit: wedge-path	Sub-orbit: $[\{c_0,c_0\},d_0]$, Orbit: triangle	95.00
Sub-orbit: $[\{c_0,c_1\},c_1]$, Orbit: triangle	Sub-orbit: $[\{a_0,d_0\},d_0]$, Orbit: triangle	95.00
Sub-orbit: $[\{c_0,c_1\},d_0]$, Orbit: triangle	Sub-orbit: $[\{d_0,d_0\},a_0]$, Orbit: triangle	95.00
Sub-orbit: $[\{c_0,c_1\},c_0]$, Orbit: triangle	Sub-orbit: $[\{d_0,d_0\},a_0]$, Orbit: triangle	95.00
Sub-orbit: $[\{c_0,c_0\},c_1]$, Orbit: triangle	Sub-orbit: $[\{d_0,d_0\},b_0]$, Orbit: triangle	95.00
Sub-orbit: $[\{c_0,c_1\},d_0]$, Orbit: triangle	Sub-orbit: $[\{a_0,d_0\},d_0]$, Orbit: triangle	95.00
Sub-orbit: $[\{c_0,c_1\},c_0]$, Orbit: triangle	Sub-orbit: $[\{c_0,c_0\},c_1]$, Orbit: triangle	95.00
Sub-orbit: $[\{b_0,d_0\},b_0]$, Orbit: triangle	Sub-orbit: $[\{d_0,d_0\},b_0]$, Orbit: triangle	94.00
Sub-orbit: $[\{a_0,d_0\},a_0]$, Orbit: triangle	Sub-orbit: $[\{c_0,d_0\},b_0]$, Orbit: triangle	94.00
Sub-orbit: $[\{c_0,d_0\},c_0]$, Orbit: triangle	Sub-orbit: $[\{c_0,c_0\},c_1]$, Orbit: triangle	94.00
Sub-orbit: $\{b_0,d_0\}$, Orbit: wedge-star	Sub-orbit: $[\{b_0,d_0\},b_1]$, Orbit: triangle	94.00
Sub-orbit: $[d_0,c_0]$, Orbit: wedge-path	Sub-orbit: $[\{a_0,d_0\},c_0]$, Orbit: triangle	94.00
Sub-orbit: $\{a_0,d_0\}$, Orbit: wedge-star	Sub-orbit: $\{d_0,d_0\}$, Orbit: wedge-star	94.00
Sub-orbit: $[\{a_0,d_0\},c_0]$, Orbit: triangle	Sub-orbit: $[\{b_0,d_0\},b_1]$, Orbit: triangle	94.00
Sub-orbit: $\{c_0,d_0\}$, Orbit: wedge-star	Sub-orbit: $[\{a_0,d_0\},a_1]$, Orbit: triangle	94.00
Sub-orbit: $[d_0,d_0]$, Orbit: wedge-path	Sub-orbit: $[\{d_0,d_0\},b_0]$, Orbit: triangle	94.00
Sub-orbit: $[\{c_0,c_0\},d_0]$, Orbit: triangle	Sub-orbit: $[\{c_0,c_0\},c_1]$, Orbit: triangle	94.00
Sub-orbit: $[\{c_0,d_0\},c_1]$, Orbit: triangle	Sub-orbit: $[\{b_0,d_0\},b_0]$, Orbit: triangle	94.00
Sub-orbit: d_0, Orbit: degree	Sub-orbit: $[d_0,b_0]$, Orbit: wedge-path	94.00
Sub-orbit: $[d_0,d_0]$, Orbit: wedge-path	Sub-orbit: $[d_0,c_0]$, Orbit: wedge-path	94.00
Sub-orbit: $[\{a_0,d_0\},c_0]$, Orbit: triangle	Sub-orbit: $[\{a_0,d_0\},a_0]$, Orbit: triangle	94.00
Sub-orbit: $[d_0,d_0]$, Orbit: wedge-path	Sub-orbit: $[\{c_0,d_0\},c_0]$, Orbit: triangle	94.00
Sub-orbit: $[\{b_0,d_0\},b_0]$, Orbit: triangle	Sub-orbit: $[\{b_0,d_0\},b_1]$, Orbit: triangle	94.00
Sub-orbit: $[d_0,c_0]$, Orbit: wedge-path	Sub-orbit: $[\{a_0,d_0\},b_0]$, Orbit: triangle	94.00
Sub-orbit: $\{b_0,d_0\}$, Orbit: wedge-star	Sub-orbit: $\{d_0,d_0\}$, Orbit: wedge-star	94.00
Sub-orbit: $[\{d_0,d_0\},d_0]$, Orbit: triangle	Sub-orbit: $[\{b_0,d_0\},b_0]$, Orbit: triangle	93.00
Sub-orbit: $\{d_0,d_0\}$, Orbit: wedge-star	Sub-orbit: $[\{b_0,d_0\},b_0]$, Orbit: triangle	93.00
Sub-orbit: $[a_0,a_0]$, Orbit: wedge-path	Sub-orbit: $[a_0,b_0]$, Orbit: wedge-path	93.00
Sub-orbit: $[d_0,a_0]$, Orbit: wedge-path	Sub-orbit: $[\{a_0,d_0\},a_0]$, Orbit: triangle	93.00
Sub-orbit: $[\{a_0,d_0\},a_0]$, Orbit: triangle	Sub-orbit: $[\{b_0,d_0\},c_0]$, Orbit: triangle	93.00
Sub-orbit: $[d_0,d_0]$, Orbit: wedge-path	Sub-orbit: $[\{c_0,d_0\},c_1]$, Orbit: triangle	93.00
Sub-orbit: $\{d_0,d_0\}$, Orbit: wedge-star	Sub-orbit: $[\{a_0,d_0\},d_0]$, Orbit: triangle	93.00
Sub-orbit: $[d_0,d_0]$, Orbit: wedge-path	Sub-orbit: $[\{b_0,d_0\},d_0]$, Orbit: triangle	93.00
Sub-orbit: $[\{d_0,d_0\},d_0]$, Orbit: triangle	Sub-orbit: $[\{b_0,b_0\},d_0]$, Orbit: triangle	93.00
Sub-orbit: $\{c_0,d_0\}$, Orbit: wedge-star	Sub-orbit: $[\{b_0,d_0\},b_1]$, Orbit: triangle	93.00
Sub-orbit: $\{a_0,a_1\}$, Orbit: wedge-star	Sub-orbit: $[\{a_0,b_0\},a_1]$, Orbit: triangle	93.00
Sub-orbit: $[d_0,d_0]$, Orbit: wedge-path	Sub-orbit: $[\{c_0,c_1\},d_0]$, Orbit: triangle	93.00
Sub-orbit: $[\{b_0,d_0\},b_0]$, Orbit: triangle	Sub-orbit: $[\{b_0,b_0\},d_0]$, Orbit: triangle	93.00
Sub-orbit: $\{d_0,d_0\}$, Orbit: wedge-star	Sub-orbit: $[\{c_0,c_1\},d_0]$, Orbit: triangle	93.00
Sub-orbit: d_0, Orbit: degree	Sub-orbit: $[\{c_0,d_0\},b_0]$, Orbit: triangle	93.00
Sub-orbit: $[d_0,d_0]$, Orbit: wedge-path	Sub-orbit: $[\{c_0,c_1\},c_0]$, Orbit: triangle	93.00
Sub-orbit: $[\{a_0,c_0\},b_0]$, Orbit: triangle	Sub-orbit: $[\{a_0,c_0\},a_1]$, Orbit: triangle	93.00
Sub-orbit: b_0, Orbit: degree	Sub-orbit: $[\{b_0,b_0\},a_0]$, Orbit: triangle	93.00
Sub-orbit: $\{d_0,d_0\}$, Orbit: wedge-star	Sub-orbit: $[\{c_0,c_1\},c_0]$, Orbit: triangle	93.00
Sub-orbit: $[d_0,d_0]$, Orbit: wedge-path	Sub-orbit: $[\{d_0,d_0\},a_0]$, Orbit: triangle	93.00
Sub-orbit: $[d_0,d_0]$, Orbit: wedge-path	Sub-orbit: $[\{c_0,c_1\},c_2]$, Orbit: triangle	93.00
Sub-orbit: $\{d_0,d_0\}$, Orbit: wedge-star	Sub-orbit: $[\{c_0,c_1\},c_1]$, Orbit: triangle	93.00
Sub-orbit: $[\{b_0,d_0\},b_0]$, Orbit: triangle	Sub-orbit: $[\{d_0,d_0\},a_0]$, Orbit: triangle	93.00

Sub-orbit: $\{d_0,d_0\}$, Orbit: wedge-star	Sub-orbit: $[\{c_0,c_1\},c_2]$, Orbit: triangle	93.00
Sub-orbit: $[\{c_0,d_0\},c_0]$, Orbit: triangle	Sub-orbit: $[\{b_0,d_0\},b_0]$, Orbit: triangle	93.00
Sub-orbit: $[d_0,d_0]$, Orbit: wedge-path	Sub-orbit: $[\{a_0,d_0\},d_0]$, Orbit: triangle	93.00
Sub-orbit: $\{a_0,d_0\}$, Orbit: wedge-star	Sub-orbit: $[\{c_0,d_0\},b_0]$, Orbit: triangle	92.00
Sub-orbit: $[\{b_0,b_0\},d_0]$, Orbit: triangle	Sub-orbit: $[\{d_0,d_0\},c_0]$, Orbit: triangle	92.00
Sub-orbit: $[a_0,a_1]$, Orbit: wedge-path	Sub-orbit: $[a_0,b_0]$, Orbit: wedge-path	92.00
Sub-orbit: $[\{c_0,c_0\},c_1]$, Orbit: triangle	Sub-orbit: $[\{d_0,d_0\},a_0]$, Orbit: triangle	92.00
Sub-orbit: $[\{c_0,c_0\},c_1]$, Orbit: triangle	Sub-orbit: $[\{c_0,c_1\},c_2]$, Orbit: triangle	92.00
Sub-orbit: $[d_0,b_0]$, Orbit: wedge-path	Sub-orbit: $[\{a_0,d_0\},a_1]$, Orbit: triangle	92.00
Sub-orbit: $[\{b_0,d_0\},d_0]$, Orbit: triangle	Sub-orbit: $[\{c_0,c_0\},c_1]$, Orbit: triangle	92.00
Sub-orbit: $[\{c_0,d_0\},d_0]$, Orbit: triangle	Sub-orbit: $[\{b_0,b_0\},d_0]$, Orbit: triangle	92.00
Sub-orbit: $[\{c_0,c_0\},c_0]$, Orbit: triangle	Sub-orbit: $[\{b_0,d_0\},d_0]$, Orbit: triangle	92.00
Sub-orbit: $[d_0,d_0]$, Orbit: wedge-path	Sub-orbit: $[\{c_0,c_1\},c_1]$, Orbit: triangle	92.00
Sub-orbit: $[\{d_0,d_0\},a_0]$, Orbit: triangle	Sub-orbit: $[\{c_0,d_0\},b_0]$, Orbit: triangle	92.00
Sub-orbit: $\{c_0,d_0\}$, Orbit: wedge-star	Sub-orbit: $[\{c_0,d_0\},b_0]$, Orbit: triangle	92.00
Sub-orbit: $[\{a_0,d_0\},d_0]$, Orbit: triangle	Sub-orbit: $[\{c_0,c_0\},c_1]$, Orbit: triangle	92.00
Sub-orbit: $[\{c_0,c_0\},c_0]$, Orbit: triangle	Sub-orbit: $[\{d_0,d_0\},b_0]$, Orbit: triangle	92.00
Sub-orbit: $[\{c_0,d_0\},d_0]$, Orbit: triangle	Sub-orbit: $[\{b_0,d_0\},b_0]$, Orbit: triangle	92.00
Sub-orbit: $\{c_0,d_0\}$, Orbit: wedge-star	Sub-orbit: $[\{a_0,d_0\},a_0]$, Orbit: triangle	92.00
Sub-orbit: $[\{c_0,d_0\},c_0]$, Orbit: triangle	Sub-orbit: $[\{b_0,b_0\},d_0]$, Orbit: triangle	92.00
Sub-orbit: $[\{b_0,d_0\},b_0]$, Orbit: triangle	Sub-orbit: $[\{a_0,d_0\},a_0]$, Orbit: triangle	92.00
Sub-orbit: $[\{b_0,d_0\},b_0]$, Orbit: triangle	Sub-orbit: $[\{c_0,c_0\},d_0]$, Orbit: triangle	92.00
Sub-orbit: $[\{b_0,b_0\},d_0]$, Orbit: triangle	Sub-orbit: $[\{c_0,c_0\},d_0]$, Orbit: triangle	92.00
Sub-orbit: $[\{c_0,c_1\},d_0]$, Orbit: triangle	Sub-orbit: $[\{c_0,c_0\},c_1]$, Orbit: triangle	92.00
Sub-orbit: $[\{c_0,d_0\},c_1]$, Orbit: triangle	Sub-orbit: $[\{c_0,c_0\},c_1]$, Orbit: triangle	92.00
Sub-orbit: $[\{b_0,b_0\},d_0]$, Orbit: triangle	Sub-orbit: $[\{b_0,d_0\},d_0]$, Orbit: triangle	91.00
Sub-orbit: $[\{b_0,d_0\},b_0]$, Orbit: triangle	Sub-orbit: $[\{a_0,d_0\},d_0]$, Orbit: triangle	91.00
Sub-orbit: $[\{b_0,d_0\},b_0]$, Orbit: triangle	Sub-orbit: $[\{b_0,d_0\},d_0]$, Orbit: triangle	91.00
Sub-orbit: $\{a_0,b_0\}$, Orbit: wedge-star	Sub-orbit: $\{b_0,b_1\}$, Orbit: wedge-star	91.00
Sub-orbit: $[\{c_0,c_1\},c_0]$, Orbit: triangle	Sub-orbit: $[\{c_0,c_0\},c_0]$, Orbit: triangle	91.00
Sub-orbit: $[\{b_0,d_0\},b_0]$, Orbit: triangle	Sub-orbit: $[\{c_0,d_0\},b_0]$, Orbit: triangle	91.00
Sub-orbit: $[\{a_0,d_0\},a_1]$, Orbit: triangle	Sub-orbit: $[\{b_0,d_0\},c_0]$, Orbit: triangle	91.00
Sub-orbit: $\{a_0,c_0\}$, Orbit: wedge-star	Sub-orbit: $[\{a_0,c_0\},a_0]$, Orbit: triangle	91.00
Sub-orbit: $[\{b_0,d_0\},b_0]$, Orbit: triangle	Sub-orbit: $[\{b_0,d_0\},c_0]$, Orbit: triangle	91.00
Sub-orbit: $\{b_0,b_1\}$, Orbit: wedge-star	Sub-orbit: $[\{b_0,b_1\},a_0]$, Orbit: triangle	91.00
Sub-orbit: $[\{c_0,d_0\},b_0]$, Orbit: triangle	Sub-orbit: $[\{b_0,d_0\},c_0]$, Orbit: triangle	91.00
Sub-orbit: $\{a_0,a_1\}$, Orbit: wedge-star	Sub-orbit: $\{a_0,a_0\}$, Orbit: wedge-star	91.00
Sub-orbit: $\{d_0,d_0\}$, Orbit: wedge-star	Sub-orbit: $[\{c_0,d_0\},b_0]$, Orbit: triangle	91.00
Sub-orbit: $\{d_0,d_0\}$, Orbit: wedge-star	Sub-orbit: $[\{c_0,c_0\},c_1]$, Orbit: triangle	91.00
Sub-orbit: $[\{b_0,b_0\},d_0]$, Orbit: triangle	Sub-orbit: $[\{d_0,d_0\},b_0]$, Orbit: triangle	91.00
Sub-orbit: $\{a_0,d_0\}$, Orbit: wedge-star	Sub-orbit: $[\{a_0,d_0\},b_0]$, Orbit: triangle	91.00
Sub-orbit: $[\{c_0,c_1\},d_0]$, Orbit: triangle	Sub-orbit: $[\{b_0,d_0\},b_0]$, Orbit: triangle	90.00
Sub-orbit: $[\{a_0,d_0\},d_0]$, Orbit: triangle	Sub-orbit: $[\{c_0,c_0\},c_0]$, Orbit: triangle	90.00
Sub-orbit: $[\{a_0,d_0\},b_0]$, Orbit: triangle	Sub-orbit: $[\{b_0,d_0\},c_0]$, Orbit: triangle	90.00
Sub-orbit: $[\{c_0,c_1\},d_0]$, Orbit: triangle	Sub-orbit: $[\{b_0,b_0\},d_0]$, Orbit: triangle	90.00
Sub-orbit: $\{d_0,d_0\}$, Orbit: wedge-star	Sub-orbit: $[\{c_0,d_0\},a_0]$, Orbit: triangle	90.00
Sub-orbit: $[\{b_0,c_0\},a_0]$, Orbit: triangle	Sub-orbit: $[\{a_0,c_0\},a_0]$, Orbit: triangle	90.00
Sub-orbit: $[\{b_0,b_0\},d_0]$, Orbit: triangle	Sub-orbit: $[\{a_0,d_0\},d_0]$, Orbit: triangle	90.00
Sub-orbit: $[d_0,b_0]$, Orbit: wedge-path	Sub-orbit: $[\{a_0,d_0\},a_0]$, Orbit: triangle	90.00
Sub-orbit: $[\{c_0,c_0\},c_0]$, Orbit: triangle	Sub-orbit: $[\{d_0,d_0\},a_0]$, Orbit: triangle	90.00
Sub-orbit: $[d_0,d_0]$, Orbit: wedge-path	Sub-orbit: $[\{c_0,c_0\},c_1]$, Orbit: triangle	90.00
Sub-orbit: $[\{b_0,d_0\},b_0]$, Orbit: triangle	Sub-orbit: $[\{a_0,d_0\},a_1]$, Orbit: triangle	90.00
Sub-orbit: $[\{b_0,b_0\},d_0]$, Orbit: triangle	Sub-orbit: $[\{d_0,d_0\},a_0]$, Orbit: triangle	90.00
Sub-orbit: $\{a_0,a_1\}$, Orbit: wedge-star	Sub-orbit: $[\{a_0,a_1\},a_0]$, Orbit: triangle	90.00
Sub-orbit: $[d_0,d_0]$, Orbit: wedge-path	Sub-orbit: $[\{b_0,d_0\},b_0]$, Orbit: triangle	90.00

Sub-orbit: $[\{c_0,d_0\},c_1]$, Orbit: triangle	Sub-orbit: $[\{b_0,b_0\},d_0]$, Orbit: triangle	90.00
Sub-orbit: $[\{a_0,d_0\},c_0]$, Orbit: triangle	Sub-orbit: $[\{b_0,d_0\},c_0]$, Orbit: triangle	90.00
Sub-orbit: $[\{c_0,c_1\},c_0]$, Orbit: triangle	Sub-orbit: $[\{b_0,b_0\},d_0]$, Orbit: triangle	90.00
Sub-orbit: $[\{c_0,c_1\},c_0]$, Orbit: triangle	Sub-orbit: $[\{b_0,d_0\},b_0]$, Orbit: triangle	90.00
Sub-orbit: $[\{c_0,c_1\},d_0]$, Orbit: triangle	Sub-orbit: $[\{c_0,c_0\},c_0]$, Orbit: triangle	89.00
Sub-orbit: $[\{c_0,c_1\},c_1]$, Orbit: triangle	Sub-orbit: $[\{c_0,c_0\},c_0]$, Orbit: triangle	89.00
Sub-orbit: c_0, Orbit: degree	Sub-orbit: $[c_0,a_0]$, Orbit: wedge-path	89.00
Sub-orbit: $\{d_0,d_0\}$, Orbit: wedge-star	Sub-orbit: $[\{b_0,b_0\},d_0]$, Orbit: triangle	89.00
Sub-orbit: $[\{c_0,d_0\},c_1]$, Orbit: triangle	Sub-orbit: $[\{c_0,c_0\},c_0]$, Orbit: triangle	89.00
Sub-orbit: $[d_0,a_0]$, Orbit: wedge-path	Sub-orbit: $[\{b_0,d_0\},b_1]$, Orbit: triangle	89.00
Sub-orbit: $[\{c_0,c_0\},c_0]$, Orbit: triangle	Sub-orbit: $[\{c_0,c_1\},c_2]$, Orbit: triangle	89.00
Sub-orbit: $\{a_0,a_1\}$, Orbit: wedge-star	Sub-orbit: $[\{a_0,a_1\},a_1]$, Orbit: triangle	88.00
Sub-orbit: $\{b_0,d_0\}$, Orbit: wedge-star	Sub-orbit: $[\{c_0,d_0\},b_0]$, Orbit: triangle	88.00
Sub-orbit: $[\{b_0,d_0\},b_0]$, Orbit: triangle	Sub-orbit: $[\{c_0,c_1\},c_2]$, Orbit: triangle	88.00
Sub-orbit: $[\{b_0,b_0\},d_0]$, Orbit: triangle	Sub-orbit: $[\{c_0,c_1\},c_2]$, Orbit: triangle	88.00
Sub-orbit: $[\{d_0,d_0\},a_0]$, Orbit: triangle	Sub-orbit: $[\{c_0,d_0\},a_0]$, Orbit: triangle	88.00
Sub-orbit: $[d_0,d_0]$, Orbit: wedge-path	Sub-orbit: $[\{b_0,b_0\},d_0]$, Orbit: triangle	88.00
Sub-orbit: $[\{d_0,d_0\},b_0]$, Orbit: triangle	Sub-orbit: $[\{c_0,d_0\},b_0]$, Orbit: triangle	88.00
Sub-orbit: a_0, Orbit: degree	Sub-orbit: $[\{a_0,b_0\},a_0]$, Orbit: triangle	87.00
Sub-orbit: $[\{c_0,c_1\},c_1]$, Orbit: triangle	Sub-orbit: $[\{b_0,d_0\},b_0]$, Orbit: triangle	87.00
Sub-orbit: $[\{c_0,c_1\},c_1]$, Orbit: triangle	Sub-orbit: $[\{b_0,b_0\},d_0]$, Orbit: triangle	87.00
Sub-orbit: d_0, Orbit: degree	Sub-orbit: $[d_0,c_0]$, Orbit: wedge-path	87.00
Sub-orbit: $[\{b_0,d_0\},a_0]$, Orbit: triangle	Sub-orbit: $[\{b_0,d_0\},b_0]$, Orbit: triangle	86.00
Sub-orbit: $\{d_0,d_0\}$, Orbit: wedge-star	Sub-orbit: $[\{c_0,c_0\},c_0]$, Orbit: triangle	86.00
Sub-orbit: a_0, Orbit: degree	Sub-orbit: $[\{a_0,b_0\},a_1]$, Orbit: triangle	86.00
Sub-orbit: $[d_0,d_0]$, Orbit: wedge-path	Sub-orbit: $[\{c_0,c_0\},c_0]$, Orbit: triangle	86.00
Sub-orbit: $[d_0,a_0]$, Orbit: wedge-path	Sub-orbit: $[\{b_0,d_0\},a_0]$, Orbit: triangle	86.00
Sub-orbit: b_0, Orbit: degree	Sub-orbit: $\{b_0,b_1\}$, Orbit: wedge-star	85.00
Sub-orbit: $[\{b_0,d_0\},b_0]$, Orbit: triangle	Sub-orbit: $[\{c_0,d_0\},a_0]$, Orbit: triangle	85.00
Sub-orbit: $[\{c_0,d_0\},c_1]$, Orbit: triangle	Sub-orbit: $[\{c_0,d_0\},b_0]$, Orbit: triangle	85.00
Sub-orbit: $[\{a_0,a_1\},a_1]$, Orbit: triangle	Sub-orbit: $[\{a_0,a_1\},b_0]$, Orbit: triangle	85.00
Sub-orbit: $\{c_0,d_0\}$, Orbit: wedge-star	Sub-orbit: $[\{d_0,d_0\},a_0]$, Orbit: triangle	85.00
Sub-orbit: a_0, Orbit: degree	Sub-orbit: $[\{a_0,a_1\},b_0]$, Orbit: triangle	85.00
Sub-orbit: d_0, Orbit: degree	Sub-orbit: $[\{b_0,d_0\},c_0]$, Orbit: triangle	85.00
Sub-orbit: $\{a_0,a_1\}$, Orbit: wedge-star	Sub-orbit: $[\{a_0,a_1\},a_2]$, Orbit: triangle	85.00
Sub-orbit: $[d_0,c_0]$, Orbit: wedge-path	Sub-orbit: $[\{a_0,d_0\},a_1]$, Orbit: triangle	85.00
Sub-orbit: $\{a_0,a_1\}$, Orbit: wedge-star	Sub-orbit: $[\{a_0,b_0\},a_0]$, Orbit: triangle	85.00
Sub-orbit: $[\{d_0,d_0\},a_0]$, Orbit: triangle	Sub-orbit: $[\{b_0,d_0\},b_1]$, Orbit: triangle	85.00
Sub-orbit: $\{a_0,b_0\}$, Orbit: wedge-star	Sub-orbit: $\{a_0,a_0\}$, Orbit: wedge-star	84.00
Sub-orbit: $[\{d_0,d_0\},a_0]$, Orbit: triangle	Sub-orbit: $[\{b_0,d_0\},c_0]$, Orbit: triangle	84.00
Sub-orbit: $[\{a_0,c_0\},a_0]$, Orbit: triangle	Sub-orbit: $[\{a_0,c_0\},b_0]$, Orbit: triangle	84.00
Sub-orbit: $[\{b_0,d_0\},a_0]$, Orbit: triangle	Sub-orbit: $[\{b_0,d_0\},c_0]$, Orbit: triangle	84.00
Sub-orbit: b_0, Orbit: degree	Sub-orbit: $[b_0,a_0]$, Orbit: wedge-path	84.00
Sub-orbit: $\{d_0,d_0\}$, Orbit: wedge-star	Sub-orbit: $[\{b_0,d_0\},c_0]$, Orbit: triangle	83.00
Sub-orbit: $[\{b_0,d_0\},a_0]$, Orbit: triangle	Sub-orbit: $[\{a_0,d_0\},c_0]$, Orbit: triangle	83.00
Sub-orbit: $\{b_0,d_0\}$, Orbit: wedge-star	Sub-orbit: $[\{a_0,d_0\},b_0]$, Orbit: triangle	83.00
Sub-orbit: $[\{c_0,d_0\},c_1]$, Orbit: triangle	Sub-orbit: $[\{b_0,d_0\},b_1]$, Orbit: triangle	83.00
Sub-orbit: $[\{a_0,d_0\},c_0]$, Orbit: triangle	Sub-orbit: $[\{c_0,d_0\},a_0]$, Orbit: triangle	83.00
Sub-orbit: $[d_0,d_0]$, Orbit: wedge-path	Sub-orbit: $[d_0,b_0]$, Orbit: wedge-path	83.00
Sub-orbit: $\{d_0,d_0\}$, Orbit: wedge-star	Sub-orbit: $[\{b_0,d_0\},b_1]$, Orbit: triangle	83.00
Sub-orbit: d_0, Orbit: degree	Sub-orbit: $[\{b_0,d_0\},b_0]$, Orbit: triangle	83.00
Sub-orbit: $[\{c_0,c_0\},c_0]$, Orbit: triangle	Sub-orbit: $[\{c_0,c_0\},c_1]$, Orbit: triangle	83.00
Sub-orbit: $[b_0,a_0]$, Orbit: wedge-path	Sub-orbit: $[b_0,b_1]$, Orbit: wedge-path	83.00
Sub-orbit: $[b_0,a_0]$, Orbit: wedge-path	Sub-orbit: $[b_0,b_0]$, Orbit: wedge-path	83.00
Sub-orbit: $[\{a_0,d_0\},b_0]$, Orbit: triangle	Sub-orbit: $[\{c_0,d_0\},a_0]$, Orbit: triangle	83.00

Sub-orbit: $[d_0,c_0]$, Orbit: wedge-path	Sub-orbit: $[\{a_0,d_0\},a_0]$, Orbit: triangle	83.00
Sub-orbit: $\{b_0,c_0\}$, Orbit: wedge-star	Sub-orbit: $[\{a_0,c_0\},a_1]$, Orbit: triangle	83.00
Sub-orbit: $[\{b_0,d_0\},b_0]$, Orbit: triangle	Sub-orbit: $[\{c_0,c_0\},c_1]$, Orbit: triangle	82.00
Sub-orbit: b_0, Orbit: degree	Sub-orbit: $[\{b_0,b_1\},a_0]$, Orbit: triangle	82.00
Sub-orbit: $[\{b_0,b_0\},d_0]$, Orbit: triangle	Sub-orbit: $[\{c_0,c_0\},c_1]$, Orbit: triangle	81.00
Sub-orbit: $[\{b_0,d_0\},b_0]$, Orbit: triangle	Sub-orbit: $[\{a_0,d_0\},b_0]$, Orbit: triangle	81.00
Sub-orbit: $[\{d_0,d_0\},b_0]$, Orbit: triangle	Sub-orbit: $[\{b_0,d_0\},c_0]$, Orbit: triangle	81.00
Sub-orbit: $[\{b_0,d_0\},d_0]$, Orbit: triangle	Sub-orbit: $[\{b_0,d_0\},c_0]$, Orbit: triangle	81.00
Sub-orbit: $[\{b_0,b_1\},a_0]$, Orbit: triangle	Sub-orbit: $[\{a_0,b_0\},a_1]$, Orbit: triangle	81.00
Sub-orbit: $[d_0,b_0]$, Orbit: wedge-path	Sub-orbit: $[\{b_0,d_0\},b_1]$, Orbit: triangle	81.00
Sub-orbit: $\{b_0,b_1\}$, Orbit: wedge-star	Sub-orbit: $[\{a_0,b_0\},a_1]$, Orbit: triangle	81.00
Sub-orbit: $[\{c_0,d_0\},c_1]$, Orbit: triangle	Sub-orbit: $[\{b_0,d_0\},c_0]$, Orbit: triangle	81.00
Sub-orbit: $[\{c_0,d_0\},a_0]$, Orbit: triangle	Sub-orbit: $[\{b_0,d_0\},c_0]$, Orbit: triangle	80.00
Sub-orbit: $[\{b_0,d_0\},b_0]$, Orbit: triangle	Sub-orbit: $[\{a_0,d_0\},c_0]$, Orbit: triangle	80.00
Sub-orbit: $[\{d_0,d_0\},c_0]$, Orbit: triangle	Sub-orbit: $[\{c_0,d_0\},b_0]$, Orbit: triangle	80.00
Sub-orbit: $\{c_0,c_1\}$, Orbit: wedge-star	Sub-orbit: $[\{c_0,c_1\},a_0]$, Orbit: triangle	80.00
Sub-orbit: $[\{d_0,d_0\},c_0]$, Orbit: triangle	Sub-orbit: $[\{b_0,d_0\},c_0]$, Orbit: triangle	80.00
Sub-orbit: $[\{a_0,d_0\},b_0]$, Orbit: triangle	Sub-orbit: $[\{c_0,d_0\},b_0]$, Orbit: triangle	80.00
Sub-orbit: $[d_0,d_0]$, Orbit: wedge-path	Sub-orbit: $[\{b_0,d_0\},c_0]$, Orbit: triangle	80.00
Sub-orbit: $[\{a_0,d_0\},d_0]$, Orbit: triangle	Sub-orbit: $[\{b_0,d_0\},c_0]$, Orbit: triangle	80.00
Sub-orbit: $[\{c_0,d_0\},c_0]$, Orbit: triangle	Sub-orbit: $[\{c_0,d_0\},b_0]$, Orbit: triangle	79.00
Sub-orbit: $[\{c_0,d_0\},c_0]$, Orbit: triangle	Sub-orbit: $[\{b_0,d_0\},c_0]$, Orbit: triangle	79.00
Sub-orbit: $[\{b_0,d_0\},d_0]$, Orbit: triangle	Sub-orbit: $[\{c_0,d_0\},b_0]$, Orbit: triangle	79.00
Sub-orbit: $\{d_0,d_0\}$, Orbit: wedge-star	Sub-orbit: $[\{b_0,d_0\},a_0]$, Orbit: triangle	79.00
Sub-orbit: $[\{a_0,d_0\},d_0]$, Orbit: triangle	Sub-orbit: $[\{c_0,d_0\},b_0]$, Orbit: triangle	78.00
Sub-orbit: $[\{d_0,d_0\},b_0]$, Orbit: triangle	Sub-orbit: $[\{b_0,d_0\},b_1]$, Orbit: triangle	78.00
Sub-orbit: $[\{a_0,d_0\},c_0]$, Orbit: triangle	Sub-orbit: $[\{c_0,d_0\},b_0]$, Orbit: triangle	78.00
Sub-orbit: $[\{c_0,d_0\},d_0]$, Orbit: triangle	Sub-orbit: $[\{c_0,d_0\},b_0]$, Orbit: triangle	78.00
Sub-orbit: $[\{a_0,b_0\},b_1]$, Orbit: triangle	Sub-orbit: $[\{a_0,b_0\},a_1]$, Orbit: triangle	78.00
Sub-orbit: $[\{b_0,c_0\},d_0]$, Orbit: triangle	Sub-orbit: $[\{b_0,d_0\},c_0]$, Orbit: triangle	77.00
Sub-orbit: $[\{c_0,d_0\},d_0]$, Orbit: triangle	Sub-orbit: $[\{b_0,d_0\},c_0]$, Orbit: triangle	77.00
Sub-orbit: $[\{b_0,c_0\},d_0]$, Orbit: triangle	Sub-orbit: $[\{c_0,d_0\},d_0]$, Orbit: triangle	77.00
Sub-orbit: $[d_0,d_0]$, Orbit: wedge-path	Sub-orbit: $[\{c_0,d_0\},b_0]$, Orbit: triangle	77.00
Sub-orbit: $[\{c_0,d_0\},c_0]$, Orbit: triangle	Sub-orbit: $[\{b_0,d_0\},b_1]$, Orbit: triangle	77.00
Sub-orbit: $[\{d_0,d_0\},d_0]$, Orbit: triangle	Sub-orbit: $[\{b_0,d_0\},c_0]$, Orbit: triangle	77.00
Sub-orbit: $[\{c_0,c_1\},d_0]$, Orbit: triangle	Sub-orbit: $[\{c_0,d_0\},b_0]$, Orbit: triangle	77.00
Sub-orbit: $[d_0,b_0]$, Orbit: wedge-path	Sub-orbit: $[\{b_0,d_0\},a_0]$, Orbit: triangle	77.00
Sub-orbit: $[\{a_0,a_1\},a_0]$, Orbit: triangle	Sub-orbit: $[\{a_0,a_1\},b_0]$, Orbit: triangle	77.00
Sub-orbit: $[\{c_0,c_0\},d_0]$, Orbit: triangle	Sub-orbit: $[\{c_0,d_0\},b_0]$, Orbit: triangle	77.00
Sub-orbit: $[d_0,c_0]$, Orbit: wedge-path	Sub-orbit: $[\{b_0,d_0\},b_1]$, Orbit: triangle	77.00
Sub-orbit: $[\{b_0,c_0\},d_0]$, Orbit: triangle	Sub-orbit: $[\{d_0,d_0\},c_0]$, Orbit: triangle	77.00
Sub-orbit: $[\{d_0,d_0\},d_0]$, Orbit: triangle	Sub-orbit: $[\{b_0,c_0\},d_0]$, Orbit: triangle	77.00
Sub-orbit: $[\{b_0,c_0\},d_0]$, Orbit: triangle	Sub-orbit: $[\{c_0,d_0\},b_0]$, Orbit: triangle	77.00
Sub-orbit: $[c_0,a_0]$, Orbit: wedge-path	Sub-orbit: $[\{a_0,c_0\},b_0]$, Orbit: triangle	77.00
Sub-orbit: $[\{d_0,d_0\},d_0]$, Orbit: triangle	Sub-orbit: $[\{c_0,d_0\},b_0]$, Orbit: triangle	77.00
Sub-orbit: $[\{b_0,c_0\},d_0]$, Orbit: triangle	Sub-orbit: $[\{c_0,c_1\},d_0]$, Orbit: triangle	76.00
Sub-orbit: $[\{b_0,c_0\},d_0]$, Orbit: triangle	Sub-orbit: $[\{c_0,c_0\},d_0]$, Orbit: triangle	76.00
Sub-orbit: $[\{b_0,c_0\},d_0]$, Orbit: triangle	Sub-orbit: $[\{b_0,d_0\},d_0]$, Orbit: triangle	76.00
Sub-orbit: $[\{b_0,c_0\},d_0]$, Orbit: triangle	Sub-orbit: $[\{d_0,d_0\},a_0]$, Orbit: triangle	76.00
Sub-orbit: $[\{b_0,c_0\},d_0]$, Orbit: triangle	Sub-orbit: $[\{d_0,d_0\},b_0]$, Orbit: triangle	76.00
Sub-orbit: $[d_0,c_0]$, Orbit: wedge-path	Sub-orbit: $[\{b_0,d_0\},c_0]$, Orbit: triangle	76.00
Sub-orbit: $[\{d_0,d_0\},b_0]$, Orbit: triangle	Sub-orbit: $[\{c_0,d_0\},a_0]$, Orbit: triangle	76.00
Sub-orbit: $[\{b_0,c_0\},d_0]$, Orbit: triangle	Sub-orbit: $[\{c_0,d_0\},c_0]$, Orbit: triangle	76.00
Sub-orbit: $\{d_0,d_0\}$, Orbit: wedge-star	Sub-orbit: $[\{b_0,c_0\},d_0]$, Orbit: triangle	76.00
Sub-orbit: $[\{c_0,c_0\},d_0]$, Orbit: triangle	Sub-orbit: $[\{b_0,d_0\},c_0]$, Orbit: triangle	76.00

Sub-orbit: $[\{b_0,d_0\},b_0]$, Orbit: triangle	Sub-orbit: $[\{c_0,c_0\},c_0]$, Orbit: triangle	76.00
Sub-orbit: $[\{b_0,b_0\},d_0]$, Orbit: triangle	Sub-orbit: $[\{c_0,c_0\},c_0]$, Orbit: triangle	76.00
Sub-orbit: $[\{b_0,c_0\},d_0]$, Orbit: triangle	Sub-orbit: $[\{a_0,d_0\},d_0]$, Orbit: triangle	76.00
Sub-orbit: $[d_0,a_0]$, Orbit: wedge-path	Sub-orbit: $[\{b_0,d_0\},c_0]$, Orbit: triangle	76.00
Sub-orbit: $[d_0,d_0]$, Orbit: wedge-path	Sub-orbit: $[\{b_0,c_0\},d_0]$, Orbit: triangle	76.00
Sub-orbit: $[\{b_0,c_0\},d_0]$, Orbit: triangle	Sub-orbit: $[\{c_0,c_1\},c_2]$, Orbit: triangle	75.00
Sub-orbit: $[\{c_0,c_1\},c_2]$, Orbit: triangle	Sub-orbit: $[\{c_0,d_0\},b_0]$, Orbit: triangle	75.00
Sub-orbit: $[\{b_0,d_0\},b_1]$, Orbit: triangle	Sub-orbit: $[\{d_0,d_0\},c_0]$, Orbit: triangle	75.00
Sub-orbit: $[\{c_0,c_1\},c_2]$, Orbit: triangle	Sub-orbit: $[\{b_0,d_0\},c_0]$, Orbit: triangle	75.00
Sub-orbit: $[\{c_0,c_1\},c_0]$, Orbit: triangle	Sub-orbit: $[\{b_0,d_0\},c_0]$, Orbit: triangle	75.00
Sub-orbit: $[\{c_0,c_1\},c_1]$, Orbit: triangle	Sub-orbit: $[\{b_0,c_0\},d_0]$, Orbit: triangle	75.00
Sub-orbit: $[\{c_0,c_1\},c_0]$, Orbit: triangle	Sub-orbit: $[\{c_0,d_0\},b_0]$, Orbit: triangle	75.00
Sub-orbit: $[\{c_0,c_1\},c_1]$, Orbit: triangle	Sub-orbit: $[\{c_0,d_0\},b_0]$, Orbit: triangle	75.00
Sub-orbit: $[\{b_0,c_0\},d_0]$, Orbit: triangle	Sub-orbit: $[\{c_0,c_1\},c_0]$, Orbit: triangle	75.00
Sub-orbit: $[\{c_0,c_1\},d_0]$, Orbit: triangle	Sub-orbit: $[\{b_0,d_0\},c_0]$, Orbit: triangle	75.00
Sub-orbit: $[\{c_0,d_0\},c_1]$, Orbit: triangle	Sub-orbit: $[\{b_0,c_0\},d_0]$, Orbit: triangle	75.00
Sub-orbit: $[\{c_0,c_1\},c_1]$, Orbit: triangle	Sub-orbit: $[\{b_0,d_0\},c_0]$, Orbit: triangle	75.00
Sub-orbit: $[\{b_0,d_0\},a_0]$, Orbit: triangle	Sub-orbit: $[\{d_0,d_0\},a_0]$, Orbit: triangle	75.00
Sub-orbit: $[\{b_0,d_0\},d_0]$, Orbit: triangle	Sub-orbit: $[\{b_0,d_0\},b_1]$, Orbit: triangle	74.00
Sub-orbit: $[\{a_0,d_0\},d_0]$, Orbit: triangle	Sub-orbit: $[\{b_0,d_0\},b_1]$, Orbit: triangle	74.00
Sub-orbit: $\{a_0,d_0\}$, Orbit: wedge-star	Sub-orbit: $[\{d_0,d_0\},a_0]$, Orbit: triangle	74.00
Sub-orbit: a_0, Orbit: degree	Sub-orbit: b_0, Orbit: degree	74.00
Sub-orbit: $[\{a_0,d_0\},d_0]$, Orbit: triangle	Sub-orbit: $[\{a_0,d_0\},c_0]$, Orbit: triangle	74.00
Sub-orbit: b_0, Orbit: degree	Sub-orbit: $[\{a_0,b_0\},b_1]$, Orbit: triangle	74.00
Sub-orbit: $\{b_0,d_0\}$, Orbit: wedge-star	Sub-orbit: $[\{d_0,d_0\},a_0]$, Orbit: triangle	74.00
Sub-orbit: b_0, Orbit: degree	Sub-orbit: $\{a_0,a_1\}$, Orbit: wedge-star	74.00
Sub-orbit: $[\{c_0,c_0\},c_1]$, Orbit: triangle	Sub-orbit: $[\{c_0,d_0\},b_0]$, Orbit: triangle	74.00
Sub-orbit: $[d_0,d_0]$, Orbit: wedge-path	Sub-orbit: $[\{a_0,d_0\},c_0]$, Orbit: triangle	74.00
Sub-orbit: $\{c_0,d_0\}$, Orbit: wedge-star	Sub-orbit: $[\{b_0,d_0\},b_0]$, Orbit: triangle	74.00
Sub-orbit: $\{c_0,d_0\}$, Orbit: wedge-star	Sub-orbit: $[\{d_0,d_0\},b_0]$, Orbit: triangle	73.00
Sub-orbit: $[\{b_0,d_0\},d_0]$, Orbit: triangle	Sub-orbit: $[\{b_0,b_1\},d_0]$, Orbit: triangle	73.00
Sub-orbit: $[\{b_0,b_1\},d_0]$, Orbit: triangle	Sub-orbit: $[\{d_0,d_0\},c_0]$, Orbit: triangle	73.00
Sub-orbit: $[\{c_0,d_0\},c_1]$, Orbit: triangle	Sub-orbit: $[\{b_0,b_1\},d_0]$, Orbit: triangle	73.00
Sub-orbit: $[\{b_0,b_1\},d_0]$, Orbit: triangle	Sub-orbit: $[\{b_0,d_0\},b_1]$, Orbit: triangle	73.00
Sub-orbit: $[d_0,b_0]$, Orbit: wedge-path	Sub-orbit: $[\{b_0,d_0\},c_0]$, Orbit: triangle	73.00
Sub-orbit: $[\{b_0,b_0\},d_0]$, Orbit: triangle	Sub-orbit: $[\{c_0,d_0\},b_0]$, Orbit: triangle	73.00
Sub-orbit: $[\{d_0,d_0\},a_0]$, Orbit: triangle	Sub-orbit: $[\{b_0,b_1\},d_0]$, Orbit: triangle	73.00
Sub-orbit: $[\{d_0,d_0\},d_0]$, Orbit: triangle	Sub-orbit: $[\{b_0,d_0\},b_1]$, Orbit: triangle	73.00
Sub-orbit: $[\{c_0,c_0\},d_0]$, Orbit: triangle	Sub-orbit: $[\{b_0,b_1\},d_0]$, Orbit: triangle	73.00
Sub-orbit: $[\{c_0,c_0\},d_0]$, Orbit: triangle	Sub-orbit: $[\{b_0,d_0\},b_1]$, Orbit: triangle	73.00
Sub-orbit: $[\{c_0,d_0\},c_0]$, Orbit: triangle	Sub-orbit: $[\{b_0,b_1\},d_0]$, Orbit: triangle	73.00
Sub-orbit: $[\{c_0,c_1\},d_0]$, Orbit: triangle	Sub-orbit: $[\{b_0,b_1\},d_0]$, Orbit: triangle	73.00
Sub-orbit: $[\{c_0,d_0\},d_0]$, Orbit: triangle	Sub-orbit: $[\{b_0,d_0\},b_1]$, Orbit: triangle	73.00
Sub-orbit: $[\{d_0,d_0\},b_0]$, Orbit: triangle	Sub-orbit: $[\{b_0,b_1\},d_0]$, Orbit: triangle	73.00
Sub-orbit: $[\{d_0,d_0\},d_0]$, Orbit: triangle	Sub-orbit: $[\{b_0,b_1\},d_0]$, Orbit: triangle	73.00
Sub-orbit: $[\{b_0,b_0\},d_0]$, Orbit: triangle	Sub-orbit: $[\{b_0,d_0\},c_0]$, Orbit: triangle	73.00
Sub-orbit: d_0, Orbit: degree	Sub-orbit: $\{d_0,d_0\}$, Orbit: wedge-star	73.00
Sub-orbit: $[d_0,c_0]$, Orbit: wedge-path	Sub-orbit: $[\{a_0,d_0\},d_0]$, Orbit: triangle	73.00
Sub-orbit: $\{b_0,b_1\}$, Orbit: wedge-star	Sub-orbit: $\{b_0,b_0\}$, Orbit: wedge-star	73.00
Sub-orbit: $[\{c_0,d_0\},d_0]$, Orbit: triangle	Sub-orbit: $[\{b_0,b_1\},d_0]$, Orbit: triangle	73.00
Sub-orbit: $[\{c_0,c_1\},d_0]$, Orbit: triangle	Sub-orbit: $[\{b_0,d_0\},b_1]$, Orbit: triangle	73.00
Sub-orbit: $[\{a_0,a_0\},a_1]$, Orbit: triangle	Sub-orbit: $[\{a_0,a_0\},b_0]$, Orbit: triangle	72.00
Sub-orbit: $[d_0,d_0]$, Orbit: wedge-path	Sub-orbit: $[\{b_0,b_1\},d_0]$, Orbit: triangle	72.00
Sub-orbit: $\{d_0,d_0\}$, Orbit: wedge-star	Sub-orbit: $[\{b_0,b_1\},d_0]$, Orbit: triangle	72.00
Sub-orbit: $[d_0,d_0]$, Orbit: wedge-path	Sub-orbit: $[\{b_0,d_0\},b_1]$, Orbit: triangle	72.00

Sub-orbit: $[\{b_0,c_0\},d_0]$, Orbit: triangle	Sub-orbit: $[\{b_0,b_0\},d_0]$, Orbit: triangle	72.00
Sub-orbit: $[\{b_0,c_0\},d_0]$, Orbit: triangle	Sub-orbit: $[\{b_0,d_0\},b_0]$, Orbit: triangle	72.00
Sub-orbit: $[\{b_0,c_0\},d_0]$, Orbit: triangle	Sub-orbit: $[\{c_0,c_0\},c_1]$, Orbit: triangle	72.00
Sub-orbit: $[\{a_0,d_0\},d_0]$, Orbit: triangle	Sub-orbit: $[\{b_0,b_1\},d_0]$, Orbit: triangle	72.00
Sub-orbit: $\{a_0,d_0\}$, Orbit: wedge-star	Sub-orbit: $[\{b_0,d_0\},b_0]$, Orbit: triangle	72.00
Sub-orbit: $\{a_0,d_0\}$, Orbit: wedge-star	Sub-orbit: $[\{b_0,d_0\},c_0]$, Orbit: triangle	71.00
Sub-orbit: $[d_0,d_0]$, Orbit: wedge-path	Sub-orbit: $[d_0,a_0]$, Orbit: wedge-path	71.00
Sub-orbit: $[\{c_0,c_0\},c_1]$, Orbit: triangle	Sub-orbit: $[\{b_0,d_0\},c_0]$, Orbit: triangle	71.00
Sub-orbit: $[\{a_0,b_0\},a_0]$, Orbit: triangle	Sub-orbit: $[\{a_0,b_0\},b_1]$, Orbit: triangle	71.00
Sub-orbit: $\{b_0,b_0\}$, Orbit: wedge-star	Sub-orbit: $[\{a_0,b_0\},a_1]$, Orbit: triangle	71.00
Sub-orbit: $[\{b_0,b_1\},d_0]$, Orbit: triangle	Sub-orbit: $[\{c_0,c_1\},c_2]$, Orbit: triangle	70.00
Sub-orbit: $\{a_0,d_0\}$, Orbit: wedge-star	Sub-orbit: $[\{a_0,d_0\},c_0]$, Orbit: triangle	70.00
Sub-orbit: $[\{a_0,b_0\},a_1]$, Orbit: triangle	Sub-orbit: $[\{b_0,b_0\},a_0]$, Orbit: triangle	70.00
Sub-orbit: $[\{c_0,c_1\},c_1]$, Orbit: triangle	Sub-orbit: $[\{b_0,d_0\},b_1]$, Orbit: triangle	70.00
Sub-orbit: $[\{b_0,d_0\},b_1]$, Orbit: triangle	Sub-orbit: $[\{c_0,c_1\},c_2]$, Orbit: triangle	70.00
Sub-orbit: $[\{c_0,c_1\},c_1]$, Orbit: triangle	Sub-orbit: $[\{b_0,b_1\},d_0]$, Orbit: triangle	70.00

Table 4. Frequent strong correlations for 4-plex economic trade networks

Social relations network

First Sub-orbit	Second Sub-orbit	% Expressed (of social relations)
SubOrbit: $[a_0,b_0]$, Orbit: wedge-path	SubOrbit: $\{a_0,b_0\}$, Orbit: wedge-star	100.00
SubOrbit: b_0, Orbit: degree	SubOrbit: $[\{b_0,b_0\},b_0]$, Orbit: triangle	100.00
SubOrbit: a_0, Orbit: degree	SubOrbit: $[a_0,b_0]$, Orbit: wedge-path	100.00
SubOrbit: a_0, Orbit: degree	SubOrbit: $\{a_0,b_0\}$, Orbit: wedge-star	100.00
SubOrbit: a_0, Orbit: degree	SubOrbit: $[a_0,a_0]$, Orbit: wedge-path	96.97
SubOrbit: $[a_0,a_0]$, Orbit: wedge-path	SubOrbit: $[a_0,b_0]$, Orbit: wedge-path	95.45
SubOrbit: a_0, Orbit: degree	SubOrbit: $\{a_0,a_0\}$, Orbit: wedge-star	93.94
SubOrbit: $[b_0,b_0]$, Orbit: wedge-path	SubOrbit: $\{b_0,b_0\}$, Orbit: wedge-star	89.39
SubOrbit: $[a_0,b_0]$, Orbit: wedge-path	SubOrbit: $[a_0,a_1]$, Orbit: wedge-path	83.33
SubOrbit: a_0, Orbit: degree	SubOrbit: $[a_0,a_1]$, Orbit: wedge-path	83.33
SubOrbit: a_0, Orbit: degree	SubOrbit: $\{a_0,a_1\}$, Orbit: wedge-star	81.82
SubOrbit: $[a_0,b_0]$, Orbit: wedge-path	SubOrbit: $\{a_0,a_0\}$, Orbit: wedge-star	75.76

Table 5. Frequent strong correlations for 2-plex social relations networks

First Sub-orbit	Second Sub-orbit	% Expressed (of social relations)
Sub-orbit: a_0, Orbit: degree	Sub-orbit: $[a_0, c_0]$, Orbit: wedge-path	100.00
Sub-orbit: b_0, Orbit: degree	Sub-orbit: $[b_0, c_0]$, Orbit: wedge-path	100.00
Sub-orbit: $[b_0, c_0]$, Orbit: wedge-path	Sub-orbit: $\{b_0, c_0\}$, Orbit: wedge-star	100.00
Sub-orbit: a_0, Orbit: degree	Sub-orbit: $\{a_0, c_0\}$, Orbit: wedge-star	100.00
Sub-orbit: a_0, Orbit: degree	Sub-orbit: $\{a_0, a_1\}$, Orbit: wedge-star	100.00
Sub-orbit: c_0, Orbit: degree	Sub-orbit: $[\{c_0, c_0\}, c_0]$, Orbit: triangle	100.00
Sub-orbit: a_0, Orbit: degree	Sub-orbit: $[a_0, a_1]$, Orbit: wedge-path	100.00
Sub-orbit: $[a_0, c_0]$, Orbit: wedge-path	Sub-orbit: $\{a_0, c_0\}$, Orbit: wedge-star	100.00
Sub-orbit: b_0, Orbit: degree	Sub-orbit: $\{b_0, c_0\}$, Orbit: wedge-star	100.00
Sub-orbit: a_0, Orbit: degree	Sub-orbit: $[a_0, a_0]$, Orbit: wedge-path	99.00
Sub-orbit: b_0, Orbit: degree	Sub-orbit: $[b_0, a_0]$, Orbit: wedge-path	98.00
Sub-orbit: $[a_0, a_0]$, Orbit: wedge-path	Sub-orbit: $[a_0, c_0]$, Orbit: wedge-path	96.00
Sub-orbit: $[a_0, c_0]$, Orbit: wedge-path	Sub-orbit: $[a_0, a_1]$, Orbit: wedge-path	96.00
Sub-orbit: a_0, Orbit: degree	Sub-orbit: $[a_0, b_0]$, Orbit: wedge-path	95.00
Sub-orbit: $[b_0, c_0]$, Orbit: wedge-path	Sub-orbit: $[b_0, a_0]$, Orbit: wedge-path	93.00
Sub-orbit: a_0, Orbit: degree	Sub-orbit: $\{a_0, a_0\}$, Orbit: wedge-star	92.00
Sub-orbit: $[a_0, b_0]$, Orbit: wedge-path	Sub-orbit: $[a_0, c_0]$, Orbit: wedge-path	89.00
Sub-orbit: $[a_0, c_0]$, Orbit: wedge-path	Sub-orbit: $\{a_0, a_1\}$, Orbit: wedge-star	89.00
Sub-orbit: $\{a_0, a_0\}$, Orbit: wedge-star	Sub-orbit: $\{a_0, a_1\}$, Orbit: wedge-star	88.00
Sub-orbit: b_0, Orbit: degree	Sub-orbit: $\{a_0, b_0\}$, Orbit: wedge-star	80.00
Sub-orbit: $[c_0, c_0]$, Orbit: wedge-path	Sub-orbit: $\{c_0, c_0\}$, Orbit: wedge-star	76.00
Sub-orbit: $[a_0, b_0]$, Orbit: wedge-path	Sub-orbit: $[a_0, a_1]$, Orbit: wedge-path	75.00
Sub-orbit: $[a_0, a_0]$, Orbit: wedge-path	Sub-orbit: $[a_0, b_0]$, Orbit: wedge-path	73.00
Sub-orbit: $[a_0, c_0]$, Orbit: wedge-path	Sub-orbit: $\{a_0, a_0\}$, Orbit: wedge-star	70.00

Table 6. Frequent strong correlations for 3-plex social relations networks

First Sub-orbit	Second Sub-orbit	% Expressed (of social relations)
Sub-orbit: b_0, Orbit: degree	Sub-orbit: $\{b_0, d_0\}$, Orbit: wedge-star	100.00
Sub-orbit: $[a_0, d_0]$, Orbit: wedge-path	Sub-orbit: $\{a_0, d_0\}$, Orbit: wedge-star	100.00
Sub-orbit: a_0, Orbit: degree	Sub-orbit: $\{a_0, d_0\}$, Orbit: wedge-star	100.00
Sub-orbit: d_0, Orbit: degree	Sub-orbit: $[\{d_0, d_0\}, d_0]$, Orbit: triangle	100.00
Sub-orbit: c_0, Orbit: degree	Sub-orbit: $[c_0, d_0]$, Orbit: wedge-path	100.00
Sub-orbit: a_0, Orbit: degree	Sub-orbit: $[a_0, d_0]$, Orbit: wedge-path	100.00
Sub-orbit: a_0, Orbit: degree	Sub-orbit: $\{a_0, a_1\}$, Orbit: wedge-star	100.00
Sub-orbit: c_0, Orbit: degree	Sub-orbit: $\{c_0, d_0\}$, Orbit: wedge-star	100.00
Sub-orbit: a_0, Orbit: degree	Sub-orbit: $[a_0, b_0]$, Orbit: wedge-path	100.00
Sub-orbit: a_0, Orbit: degree	Sub-orbit: $[a_0, a_1]$, Orbit: wedge-path	100.00
Sub-orbit: $[b_0, d_0]$, Orbit: wedge-path	Sub-orbit: $\{b_0, d_0\}$, Orbit: wedge-star	100.00
Sub-orbit: b_0, Orbit: degree	Sub-orbit: $[b_0, a_0]$, Orbit: wedge-path	100.00
Sub-orbit: $[a_0, a_1]$, Orbit: wedge-path	Sub-orbit: $[a_0, d_0]$, Orbit: wedge-path	100.00
Sub-orbit: $[c_0, d_0]$, Orbit: wedge-path	Sub-orbit: $\{c_0, d_0\}$, Orbit: wedge-star	100.00
Sub-orbit: $[a_0, d_0]$, Orbit: wedge-path	Sub-orbit: $[a_0, b_0]$, Orbit: wedge-path	100.00
Sub-orbit: $[a_0, d_0]$, Orbit: wedge-path	Sub-orbit: $\{a_0, a_1\}$, Orbit: wedge-star	100.00
Sub-orbit: b_0, Orbit: degree	Sub-orbit: $[b_0, d_0]$, Orbit: wedge-path	100.00
Sub-orbit: c_0, Orbit: degree	Sub-orbit: $[c_0, a_0]$, Orbit: wedge-path	99.00
Sub-orbit: $[b_0, a_0]$, Orbit: wedge-path	Sub-orbit: $[b_0, d_0]$, Orbit: wedge-path	99.00
Sub-orbit: $[a_0, a_1]$, Orbit: wedge-path	Sub-orbit: $[a_0, b_0]$, Orbit: wedge-path	99.00
Sub-orbit: a_0, Orbit: degree	Sub-orbit: $[a_0, a_0]$, Orbit: wedge-path	97.00
Sub-orbit: $[a_0, a_0]$, Orbit: wedge-path	Sub-orbit: $[a_0, b_0]$, Orbit: wedge-path	96.00
Sub-orbit: $\{a_0, a_1\}$, Orbit: wedge-star	Sub-orbit: $\{a_0, a_0\}$, Orbit: wedge-star	92.00
Sub-orbit: $[a_0, d_0]$, Orbit: wedge-path	Sub-orbit: $[a_0, a_0]$, Orbit: wedge-path	90.00
Sub-orbit: a_0, Orbit: degree	Sub-orbit: $\{a_0, a_0\}$, Orbit: wedge-star	90.00
Sub-orbit: b_0, Orbit: degree	Sub-orbit: $\{a_0, b_0\}$, Orbit: wedge-star	89.00
Sub-orbit: a_0, Orbit: degree	Sub-orbit: $[a_0, c_0]$, Orbit: wedge-path	79.00
Sub-orbit: c_0, Orbit: degree	Sub-orbit: $\{a_0, c_0\}$, Orbit: wedge-star	78.00
Sub-orbit: $[a_0, a_1]$, Orbit: wedge-path	Sub-orbit: $\{a_0, d_0\}$, Orbit: wedge-star	77.00
Sub-orbit: $[c_0, d_0]$, Orbit: wedge-path	Sub-orbit: $[c_0, a_0]$, Orbit: wedge-path	76.00

Table 7. Frequent strong correlations for 4-plex social relations networks

References

1. http://economics.mit.edu/faculty/eduflo/social
